# Coupling complementary strategy to flexible graph neural network for quick discovery of coformer in diverse co-crystal materials

**DOI:** 10.1038/s41467-021-26226-7

**Published:** 2021-10-12

**Authors:** Yuanyuan Jiang, Zongwei Yang, Jiali Guo, Hongzhen Li, Yijing Liu, Yanzhi Guo, Menglong Li, Xuemei Pu

**Affiliations:** 1 College of Chemistry, Sichuan University, Chengdu, 610064 China; 2 Institute of Chemical Materials, China Academy of Engineering Physics, Mianyang, 621900 China; 3 College of Computer Science, Sichuan University, Chengdu, 610064 China

**Keywords:** Cheminformatics, Crystal engineering, Self-assembly, Computational chemistry

## Abstract

Cocrystal engineering have been widely applied in pharmaceutical, chemistry and material fields. However, how to effectively choose coformer has been a challenging task on experiments. Here we develop a graph neural network (GNN) based deep learning framework to quickly predict formation of the cocrystal. In order to capture main driving force to crystallization from 6819 positive and 1052 negative samples reported by experiments, a feasible GNN framework is explored to integrate important prior knowledge into end-to-end learning on the molecular graph. The model is strongly validated against seven competitive models and three challenging independent test sets involving pharmaceutical cocrystals, π–π cocrystals and energetic cocrystals, exhibiting superior performance with accuracy higher than 96%, confirming its robustness and generalization. Furthermore, one new energetic cocrystal predicted is successfully synthesized, showcasing high potential of the model in practice. All the data and source codes are available at https://github.com/Saoge123/ccgnet for aiding cocrystal community.

## Abstract (web summary)

Experimental determination of new cocrystals remains challenging due to the need of a systematic screening with a large range of coformers. Here the authors develop a flexible deep learning framework based on graph neural network demonstrated to quickly predict the formation of co-crystals.

## Introduction

Cocrystals (CCs) are defined as a kind of single-phase crystalline materials composed of two or more neutral molecules assembled by noncovalent forces in definite stoichiometric ratio, which are neither solvates nor simple salts^1^. The co-crystallization could offer an opportunity to achieve novel properties for functional molecules through noncovalent bond synthesis with low cost, structural flexibility, and solution-processing capability^2^. Consequently, cocrystal engineering has been served as an effective design strategy in pharmaceutical, chemistry, and material fields. For example, CCs are used as means to address physicochemical, biopharmaceutical and mechanical properties and expand solid form diversity of Activate Pharmaceutical Ingredients (APIs)^3^. For organic functional materials, CCs have advanced optical, electrical and innovative functionalities^4^. Also, the cocrystal is an effective lever to improve the performance of explosives in order to achieve low-sensitivity and high-energy^5–8^.

Despite of the fascinating promises, how to choose coformer is a primary key in cocrystal engineering since the co-crystallization only occurs between some certain molecules^9,10^. Experimental determination of new co-crystals generally involves systematic screening with a large range of coformers, thus being costly in both time, effort and laboratory resources. To mitigate the problem, some computational approaches were proposed to predict co-formers likely to form CCs, for example, structural analysis using experimental data from the Cambridge Structural Database (CSD)^11^, network-based link prediction for cocrystal design^12^, thermodynamic characteristics of cocrystal formation^13^, molecular dynamics simulation^14^, intermolecular site pairing energy (ISPE)^15^, COSMO-RS (Conductor like Screening Model for Real Solvents) based on calculation of mixing enthalpy in a supercooled liquid phase^16^, and coformer screening based on cloud-computing crystal structure prediction (CSP) technology^17^. These methods above roughly follow knowledge-based^11–13^ and physics-based^14–17^ paradigms, which indeed provide useful guidelines for experimental designs. However, they are limited in the generalization for diversity of noncovalent interactions and molecular chemical structures. Therefore, it is highly desired to develop more general strategies with lower computation cost.

Recently, data-driven machine learning (ML) methods have become increasingly popular in chemical and material fields^18^ due to their optimization strategies that are automatically improved by empirical data from statistical perspectives, thus providing smart navigation in nearly infinite chemical space^19^. Several works already utilized the ML methods to make meaningful attempts to the cocrystal prediction, involving support vector machines (SVMs)^20^, Multivariate Adaptive Regression Splines^21^, Random Forest (RF), and Deep neural network (DNN)^22^. However, these ML methods coupled with the molecular descriptors or fingerprints only exhibited moderate accuracy for the cocrystal prediction. With rapidly accumulated data and booming of Graphic Processing Units (GPUs), deep learning (DL) has been far beyond conventional ML methods in many research domains^23–25^. In particular, graph neural networks (GNNs), a subset of DL, has received increasing attentions due to great expressive power of graphs^26^. For GNN, end-to-end learning on the molecular graph replaces traditional feature engineering to model chemical properties^27,28^, which could avoid the conformational challenge from 3D representations of compounds^29^. Very recently, one GNN-based work on the cocrystal screening were reported to achieve ~97% accuracy for validation sets and ~80% for independent test sets^30^. Despite the performance on the validation set was boosted by GNN, the prediction accuracy on the independent test set that reflects the robustness and the generalization of the ML model to unseen samples is still moderate. However, improving the generalization ability has been considered to be one of the most difficult challenges for the ML^31,32^, which involves dataset, feature representation and model algorithm.

As accepted, the data-driven MLs mainly rely on the large amount of high-quality data. CSD^33^ contains a wealth of cocrystal structures that can supports the DL, but only be restricted to the positive samples (cocrystals) while there has been lack of invalid coformer combinations (negative sample) reported. Thus, Vriza et al.^34^ only used positive samples to construct one classification model to predict π–π co-crystals. In order to construct a balanced negative samples, Devogelaer and Wang combined two coformers into invalid co-crystals with the aid of some computational ways like network-based link prediction^30^ and molecular similarity-based method^22^. In the case of experimental data unavailable, the computation way is supposed to be a good alternative. However, for the CCs, there practically have been some negative samples reported by experiments despite of sparseness with respect to the positive samples, leading to an imbalanced dataset. ML on the imbalanced dataset is easily biased towards the majority group^35,36^, thus being difficult. However, in the real world, the problem of uneven data representation is often faced. Moreover, from the data mining perspective, the minority class is the one more important, as it may carry important and useful knowledge to determine the boundary between success and failure. Utilizing failed experiments, some ML-based works already achieved successes in assisting material synthesis^37,38^.

In addition, the feature representation characterizing the sample is also a key of the ML-based model, in particular for the imbalanced data. If both classes with high disproportion are well represented with non-overlapping distributions, good classification rates are still obtained by ML-based classifiers^36^. Conventional ML algorithms generally involve feature selections or optimizations (also called hand-engineering) to improve the model performance. While modern DL methods like GNNs often follow an “end-to-end” self-learning strategy, which emphasizes minimal a priori representational and computational assumptions to avoid “hand-engineering”. In other words, DL is isolated from potentially useful knowledge^39^. Theoretically, DL models can bypass “hand-engineering” features with sufficiently large data. However, the data available are often limited in many fields, which hardly support DL to learning sufficient knowledge characterizing the target property. In this case, it should be advocated for an approach that benefits from the complementary strength of “hand-engineering” and “end-to-end” learning^40^, just as biology uses nature and nurture cooperatively.

Motivated by the challenges above, we, in this work, reconstruct a reliable co-crystal dataset composed of 7871 samples, where 1052 negative samples are all collected from experimental reports to minimize the false negative and 6819 positive samples still come from CSD. To more completely capture the main driving force to the co-crystallization from the limited and imbalanced dataset, a complementary strategy is proposed for the co-crystal representation through combining the molecular graph and 12 molecular descriptors from priori knowledge that was revealed to make important contributions to the cocrystal formation^11,41,42^. With the feature representation, we explore a flexible GNN-based DL framework that effectively integrates the empirical knowledge into end-to-end learning on the molecular graph, which can be feasibly applied to the CCs that are significantly different from the training dataset through transfer learning. We name it as Co-Crystal Graph Network (CCGNet). To sufficiently evaluate its performance, seven competitive models were adopted to compare, including two traditional MLs and five DL models. In addition, different from the previous ML works that used one type of independent test similar to the training set, the robustness and generalization of CCGNet are strongly validated against three different types of co-crystal systems (pharmaceutical CCs, π–π CCs and energetic CCs) as unseen cases, which have been considered to be challenging for virtual screening of co-crystals^30,34,43^. Our model showcases high accuracy for the three independent testing sets, outperforming the competitive models. Based on the prediction result, a new energetic co-crystal predicted is successfully synthesized, further confirming the potential of CCGNet in practical application.

## Results

### Data collection and augmentation

**Fig. 1:**
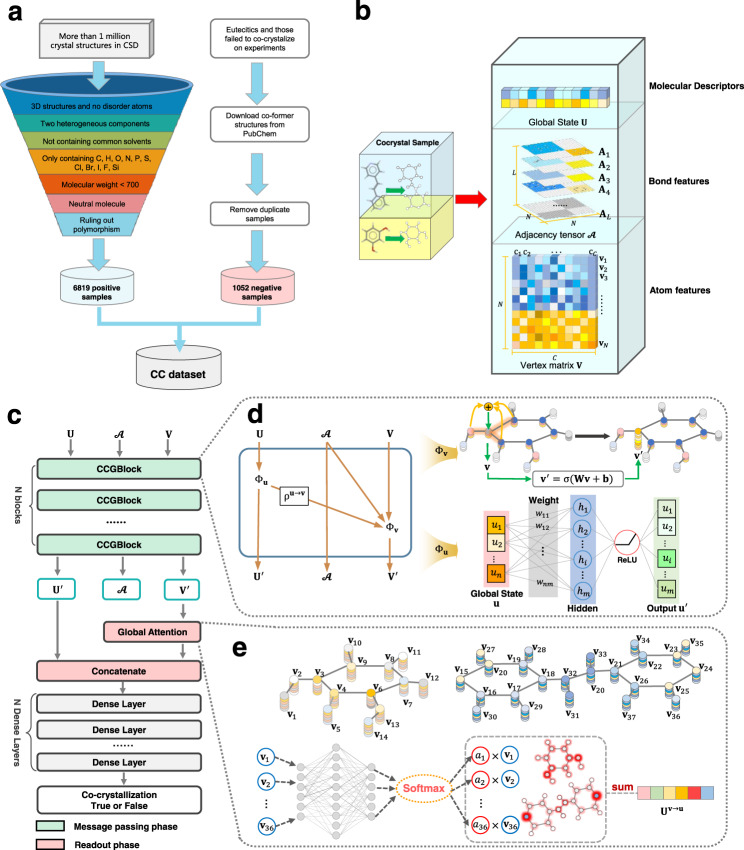
Overview of CCGNet cocrystal-screening framework. **a** The flow chart of sample collection. Left: the collection of cocrystal positive samples. Right: the collection of cocrystal negative samples. **b** Sample Representation. **c** The architecture of the CCGNet model. Green and pink denote the calculation block at the message passing phase and the readout phase, respectively. **d** Customization of CCGBlock. ${\Phi }_{{{{{{\bf{u}}}}}}}$ is the global state function realized by a single-layer feedforward neural network while ${\Phi }_{{{{{{\bf{v}}}}}}}$ is a Graph-CNN layer to propagate and update node information. ${{{{{{\rm{\rho }}}}}}}^{{{{{{\bf{u}}}}}}\to{{{{{\boldsymbol{\to }}}}}}{{{{{\bf{v}}}}}}}$ is a concatenation operation, which embeds hidden representation of ${{{{{\bf{u}}}}}}$ into atom vectors of each coformer. ${{{{{\rm{\sigma }}}}}}$ is activation function. **e** Illustration of the global attention. ${{{{{{\bf{v}}}}}}}_{i}$ is node embedding. ${a}_{i}$ is the attention weight of each node. ${{{{{{\bf{U}}}}}}}^{{{{{{\bf{v}}}}}}\to{{{{{\boldsymbol{\to }}}}}}{{{{{\bf{u}}}}}}}$ is a weighted summation of products of ${{{{{{\bf{v}}}}}}}_{i}$ and ${a}_{i}$.

Data availability is a critical bottleneck that limits applications of DL in cocrystal engineering and data quality is another key to the model performance. Thus, to obtain a reliable dataset, we construct a large dataset containing 7871 samples (called as CC dataset below), which are composed of 6819 positive samples (Supplementary Data 1) and 1052 negative ones (Supplementary Data 2). The positive samples come from CSD^33^, which contains more than one million crystal structures of small molecules and metal–organic molecular crystals resolved by X-ray and neutron diffraction experiments. As illustrated by Fig. 1a, the CCs are screened from CSD in terms of the following conditions:

1. Only containing two chemically different polyatomic units.
2. Having 3D structures and no disorder atoms to avoid low-quality structures.
3. Not containing any of a set common solvents or small molecule^9,44^, which are liquid/gaseous at room temperature, as listed in the Supplementary Table 1.
4. Only containing C, H, O, N, P, S, Cl, Br, I, F, and Si elements, ruling out metal elements.
5. Molecular weight of each component <700, considering the fact that most organic CCs are generally small molecules.
6. Being neutral components to exclude salts because most functional CCs are neutral/quasineutral^45^.
7. Ruling out polymorphism to remove duplicate samples, considering that different crystal structures can be formed between the two same co-formers when the crystallization conditions change.

Consequently, 6819 positive samples are obtained using CCDC python Application Programming Interface. Different from previous works that combined invalid co-crystal as negative samples merely using the computational rules^22,30^, our negative samples are collected from experimental reports scattered in ~186 pieces of literature (Supplementary Data 2) in order to minimize the false negative. For example, eutectics reported are taken as the negative samples since they are lack of long-range-order^46^. In addition, those that are failed to co-crystalize in the cocrystal-screening experiments are served as the negative samples. All coformer structures of the negative samples are downloaded from PubChem. Then, we use the PubChem Compound CID as the unique identification of each negative coformer to remove duplications. Taken together, we collected 1052 negative samples. The 7871 samples could support the DL training and also provides data resource for studying other properties of the CCs in the future. In addition, when the dataset is used to train the ML, we adopt a data augmentation strategy in order to enhance the robustness and the generalization ability of the model. The data augmentation strategy is usually advocated in DL^31^, in particular for the limited dataset. As the input of cocrystal involve a pair of coformers, we exchange their input orders to double the amount of the samples, in turn augmenting the dataset.

### Representation of samples

As accepted, the sample representation is essential for the ML to fit the relationship between the molecular structure and its property. Different from traditional GNNs with samples characterized only by the molecular graph, we propose a complementary feature representation by combining priori knowledge and self-learning on the molecular graph to more completely capture the main driving force to co-crystallization from the limited dataset. Table 1 lists atomic and covalent bond features for the molecular graph used in the work. Twelve molecular descriptors are selected to represent priori knowledge since they were revealed by related studies to be highly associated with the cocrystal1ization^11,41,42^. Table 2 shows the 12 selected molecular descriptors involving the molecular shape, size, polarity, flexibility, and hydrogen bond tendency, which can be quickly calculated to facilitate high-throughput screening. As depicted by Fig. 1b, we take these molecular descriptors from the domain knowledge as global state ${{{{{\bf{u}}}}}}$, which is embedded into a 2*12 matrix. The covalent bond information from the molecular graph is represented by an adjacency tensor ${{{{\boldsymbol{{{{{\mathscr{A}}}}}}}}}}$. Each slice ${{{{{{\bf{A}}}}}}}_{{{{{{\boldsymbol{l}}}}}}}$ is an adjacency matrix that represents one bond type, through which the other features besides the covalent bond also can be embedded into ${{{{\boldsymbol{{{{{\mathscr{A}}}}}}}}}}$ as extra slices. The features of the atomic level from the molecular graph are transformed to the vertex matrix.

**Table 1:** Atomic and bond attributes used in CCGNet.

Feature	Description
Atom	
Atom type	Cl, N, P, Br, B, S, I, F, C, O, H (one-hot)
Hybridization	SP2, SP3, SP, S (one-hot)
Chirality	None, R, S (binary)
is_chiral	True or False (binary)
is_spiro	True or False (binary)
is_cyclic	True or False (binary)
is_aromatic	True or False (binary)
is_acceptor	True or False (binary)
is_donor	True or False (binary)
Explicitvalence	Integer
Implicitvalence	Integer
Formal charge	Integer
Degree	Integer
Total H number	Integer
Vdw radius	Float
Atomic_number	Integer
Bond	
Bond type	Single, double, triple, aromatic (one-hot)

**Table 2:** Molecular descriptors used as the global state in CCGNet.

Molecular descriptor	Description
S	Short axis of an enclosing box (float)
S_L	S/long axis of an enclosing box (float)
S_M	S/medium axis of an enclosing box (float)
M_L	Medium axis of an enclosing box/long axis of an enclosing box (float)
Globularity	Surface of a sphere with the same volume as the molecule/area (float)
FrTPSA	TPSA/SASA (float)
Fr_NO	(n_N + n_O)/n_heavy (float)
Fr_AromaticAtoms	n_ AromaticAtom/n_heavy (float)
HBA	the number of H-bond acceptor (integer)
HBD	the number of H-bond donor (integer)
RBN	the number of rotatable bond (integer)
Dipole_Moment	Dipole moment (float)

### Construction of co-crystal graph network (CCGNet) model

With the complementary feature proposed, we accordingly construct a flexible graph neural network-based co-crystal prediction model named as CCGNet. Here, we formalize the CCGNet framework by introducing related concepts of Graph Nets (GNs)^40^ and Message Passing Neural Networks (MPNNs)^47^ paradigms. As shown in Fig. 1c, CCGNet is mainly composed of two stages, i.e., message passing phase and readout phase. The message passing is the core of MPNNs, which propagate vertex embedding to neighbors and update its embedding. As depicted by Fig. 1d, the message passing phase can be consist of N CCGBlocks (four CCGBlocks in this work), which are formalized by GN block. CCGBlock involves two trainable functions that are ${{{{{{\mathbf{\Phi }}}}}}}_{{{{{{\bf{u}}}}}}}$ and ${{{{{{\mathbf{\Phi }}}}}}}_{{{{{{\bf{v}}}}}}}$. Herein, ${{{{{{\mathbf{\Phi }}}}}}}_{{{{{{\bf{u}}}}}}}$ is defined as a global state function and is constructed by a single-layer feedforward neural network, which computes a hidden representation of the global state associated with the 12 hand-selected molecular descriptors. ${{{{{{\mathbf{\Phi }}}}}}}_{{{{{{\bf{v}}}}}}}$, a Graph-CNN layer^48^, is utilized to propagate and update information between nodes/atoms of the molecular graph using an adjacent tensor that represents the edges/bonds. ${{{{{{\mathbf{\rho }}}}}}}^{{{{{{\bf{u}}}}}}\to {{{{{\bf{v}}}}}}}$ is a concatenation operation, which is used to embed the hidden representation of ${{{{{\bf{u}}}}}}$ into the atom vector of each coformer.

In the readout phase, we also conduct the concatenate operation to further fuse the multilevel features, and introduce global attention mechanism^49^ into the readout function to calculate feature vectors from the molecular graph, which uses the weighting summation of the atom vectors instead of simply summing, as illustrated by Fig. 1e. To stabilize the learning process of self-attention and further optimize hidden embedding, we construct multi-head attention framework, which parallelly calculates $k$ independent attention coefficients of each atom to produce $k$ independent embeddings and then concatenate them to the vector for whole sample representation. After the global attention, we concatenate the hidden representation ${{{{{{\bf{U}}}}}}}^{{\prime} }$ of the global state with the graph embedding to further enrich the information. Finally, sequential dense layers are applied to the final prediction for co-crystal formation, as highlighted in the gray block in Fig. 1c. The details regarding the node update function, global state function, concatenation operation and readout function coupled with the attention mechanism are described in Methods.

### Ablation experiments on feature representation and model architecture

**Fig. 2:**
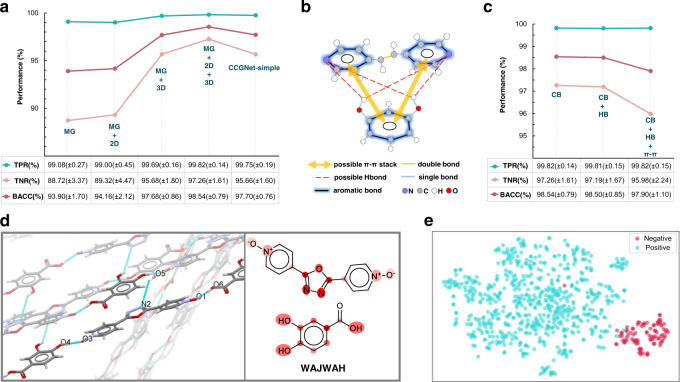
Ablation experiments on feature representation and model architecture. **a** Prediction performance for the network trained with different subsets of features and different concatenation ways for the tenfold cross-validation set. MG denotes only using Molecular Graph as input. MG + 2D denotes a combination of MG and the 2D descriptors while MG + 3D means a combination of MG and the 3D descriptors. MG + 2D + 3D represents the complementary input of MG, 2D, and 3D descriptors. CCGNet-simple denotes that the concatenation operation in each CCGBlock is removed, only retaining the concatenation at the readout phase. CCGNet-simple also uses the combination of MG, 2D and 3D descriptors as input. TPR, TNR, and BACC denote true positive rate, true negative rate and balanced accuracy (see Methods for details), respectively. **b** Illustration of two possible intermolecular interactions as new edge features for the molecular graph. The red dashes denote possible H-bonding (HB) and the yellow arrows represent the possible π–π stack (π–π). **c** Model performances of CCGNet trained with different edge representations for the tenfold cross-validation set. CB: the complementary features composed of the 12 molecular descriptors and the molecular graph only involving the covalent bond as the edge feature. CB + HB: introduction of the intermolecular H-bonding (HB) into the molecular graph of CB. CB + HB + π–π: adding HB and the intermolecular π–π interaction (π–π) into the molecular graph of CB. **d** Attention visualization for one representative cocrystal involving the intermolecular H-bonding and π–π interaction. The real co-crystal structure displayed by Mercury and the 2D structure is highlighted by the attention weights. The redder the color, the greater the attention weight. The cyan dash line denotes the intermolecular H-bonding. **e** t-SNE analysis on one representative fold of the tenfold cross-validation for CCGNet. Hidden representations are extracted after the concatenation operation in the readout phase. Red: Negative sample. Blue: Positive sample.

We conduct some ablation studies on the feature representation and the model framework to investigate whether they are essential for the model performance. For the 12 molecular descriptors, seven are correlated with the 3D conformation, such as S, S_L, M_L, S_M, Globularity, FrTPSA and Dipole_Moment, which are labeled as 3D descriptors. The remaining five descriptors can be decided by the 2D structure, thus labeled as 2D ones. We separately remove the 2D descriptors, the 3D descriptors and all the 12 molecular descriptors from the global state and then test the impacts of the remaining features. Figure 2a shows their prediction performances on the tenfold cross-validation set, where the model only using the molecular graph presents the lowest accuracy (93.90% of BACC). After including the 2D descriptors, the prediction performance is slightly improved to be 94.16%. However, substantial improvement is achieved (97.68%) by alone embedding the seven 3D descriptors into the molecular graph. When the 12 molecular descriptors are all fused into the molecular graph, the prediction accuracy is further improved to be 98.54%. The result indicates that the self-learning of GNN on the molecular graph from the limited dataset hardly grasps sufficient structure information, in particular for the features associated with the 3D conformation. In the case, the feature complementary will alleviate the limitation.

In addition, intermolecular H-bonds and π–π interactions have been considered to dominate the process of recognition and assembly for the co-crystallization^4,50,51^. Thus, we also investigate whether the prediction accuracy can be further improved by adding the two intermolecular interactions as two types of new edge features into the adjacency tensor for the molecular graph, as illustrated by Fig. 2b. Not expected, the model performance is not improved but dropping to some extent, as reflected by Fig. 2c. Practically, the 12 molecular descriptors involve the number of aromatic atoms and H-bond donors/acceptors, which are associated with the intermolecular H-bonding and π–π interaction. Furthermore, our CCGNet model also introduces the attention mechanism in the readout phase to further optimize the feature space. As reflected by Fig. 2d, a pair of coformers in the co-crystal structure exhibit –O5–H···N2, –O4–H···O3, and –O6–H···O1 H-bonding as well as π–π interaction between the benzene ring and the oxadiazole ring, while the attention weights just capture these groups involving the two intermolecular interactions. More examples and discussion are shown in Supplementary Fig. 1 and Supplementary Discussion. Therefore, the extra addition of the two edge features conversely increases the redundancy of features, making the model learning more difficult, in turn decreasing the prediction accuracy. Next, we perform an ablation experiment on the concatenation way that is crucial for the effectiveness of fusing the global state and the node feature, where we remove the concatenation (${{{{{{\rm{\rho }}}}}}}^{{{{{{\bf{u}}}}}}\to {{{{{\bf{v}}}}}}}$) in each CCGBlock and only retaining the concatenation at the readout stage (Supplementary Fig. 2). We call the framework as CCGNet-simple. It can be seen from Fig. 2a that CCGNet-Simple also get high accuracy, but still slightly lower than that including the concatenation at each CCGBlock. Finally, we use t-distributed stochastic neighbor embedding (t-SNE) analysis^52^ to visualize the input vectors of the dense layer for the tenfold cross-validation. Figure 2e representatively displays t-SNE of one-fold and the ten folds can be found in Supplementary Fig. 3. It is clear that the cocrystal embedding learned by CCGNet can well separate the positive and negative samples even in the unbalanced data distribution, which is benefited from the complementary feature and the reasonable model framework.

### Performance of CCGNet and comparison with competitive models

To assess the performance of CCGNet, we also conduct a comparison study with seven competitive models involving classical ML (SVM and RF) and DL (GNN and DNN) algorithms. Herein, SVM and RF use our twelve molecular descriptors as input. For GNN, we focus on three frameworks solely using the molecular graph as input, including GCN^30^, enn-s2s^47^, and Graph-CNN^48^. As mentioned above, GCN exhibited a high performance on the validation set but moderate performance (~80%) for the independent test set of a balanced cocrystal dataset^30^. enn-s2s is a classic GNN based on the MPNN paradigm that was summarized from many GNNs by Gilmer et al.^47^ and showcased excellent performance in predicting some quantum chemical properties, while Graph-CNN as an extension of Convolutional Neural Network (CNN) in graph data achieved good accuracy in the binary classification task for the activity of compounds against cancer cell and the categories of enzymes^48^. Despite the fact that Graph-CNN^48^ and enn-s2s^47^ were not used in the cocrystal prediction, we still take them as the competitive models in order to more comprehensively gauge our model architecture and the feature complementary with respect to the classical GNN frameworks. It is noted that we did some modifications on the output layer of enn-s2s to meet the cocrystal prediction. In addition, two DNN models are considered in the comparison. One is constructed in terms of classical DNN paradigm only using the 12 molecular descriptors as input (labeled as DNN-des), through which we could further evaluate the performance of the DL only using the features from prior knowledge. The second DNN model coupled with the extended-connectivity fingerprints^53^ (ECFP) is derived from the cocrystal-screening work^30^, which is labeled as DNN-FP. All these models are retrained on our cocrystal dataset and Bayesian optimization is used to search their optimal hyper-parameters. Details regarding the construction of the seven competitive models and their Bayesian optimizations are described in Supplementary Methods. Supplementary Tables 2, 3 list the hyper-parameter spaces, while Supplementary Fig. 5 shows the best configurations determined by Bayesian optimization for the all the models including our CCGNet.

Table 3 depicts the performances of the models on the tenfold cross-validation set coupled with the data augmentation. It can be seen that all the competitive models give high prediction accuracies for the positive samples (TPRs), higher than 98%. But, their performances (86–90%) on the negative samples (TNRs) are significantly lower than TPRs. The observation is consistent with the prevalent problem that MLs on the unbalanced dataset is generally biased to the majority group (i.e., positive samples in the work). Thus, in the case, it is required to more carefully design the ML model. As expected, our CCGNet alleviates the challenge by means of the complementary feature and the flexible architecture, thus its accuracy on the negative samples (TNR) is still high up to 97.26% even in the uneven data distribution. Consequently, BACC of our CCGNet is highest (98.54%), greatly outperforming the seven competitive models (92.52–94.46%). In addition, Supplementary Table 4 exhibits the effect of the data augmentation on the prediction performances of all the models. It can be seen that most models present variances to the different input orders of a pair of coformers before the data augmentation. However, they become insensitive to the permutation order after using the data augmentation, and the overall prediction accuracies are to different extent improved, in particular for the GCN, DNN-FP, DNN-desc, RF and SVM models. In other words, the data augmentation could endow the ML model with invariance to some interferences, thus improving its robustness and performance.

**Table 3:** Performances of the models on the tenfold cross-validation.

Model	TPR (%)	TNR (%)	BACC (%)
SVM^c^	99.11 (±0.41)	89.81 (±3.55)	94.46 (±1.85)
RF^c^	99.82 (±0.15)	87.05 (±3.87)	93.44 (±1.89)
DNN-des^c^	99.55 (±0.19)	89.11 (±2.42)	94.33 (±1.25)
DNN-FP^b,30^	98.57 (±0.46)	86.48 (±4.86)	92.52 (±2.37)
enn-s2s^a,47^	98.63 (±0.38)	89.90 (±4.98)	94.27 (±2.41)
Graph-CNN^a,48^	98.94 (±0.39)	87.20 (±3.33)	93.07 (±1.60)
GCN^a,30^	98.98 (±0.43)	87.64 (±3.47)	93.31 (±1.76)
CCGNet^d^	99.82 (±0.14)	97.26 (±1.61)	98.54 (±0.79)

^a^Model input is the molecular graph.

^b^Model input is ECFP4.

^c^Model input is the twelve molecular descriptors.

^d^Model input is a combination of the molecular graph and the twelve molecular descriptors.

### Verification and application of CCGNet

**Fig. 3:**
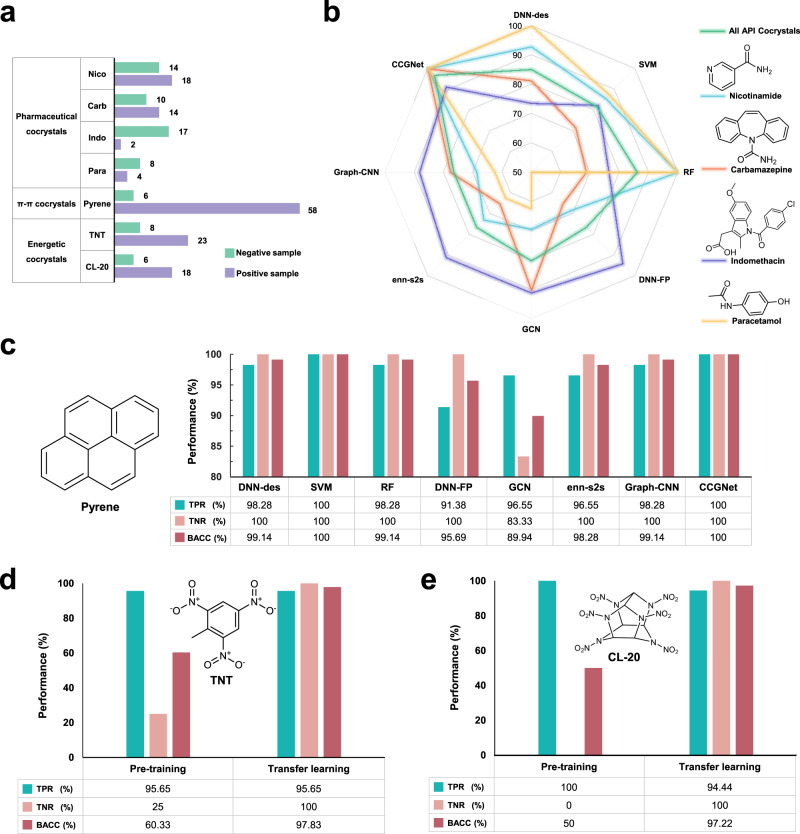
Model performances on the independent test sets. **a** Samples of the independent test sets. Nico Nicotinamide, Carb Carbamazepine, Indo Indomethacin, Para Paracetamol. **b** The balanced accuracy for the four APIs. Cyan Nicotinamide, Orange Carbamazepine, Violet Indomethacin, Yellow Paracetamol, Green holistic performance of all APIs. **c** The prediction performance on the pyrene cocrystals. TPR, TNR, and BACC denote true positive rate, true negative rate and balanced accuracy, respectively. **d** The prediction performance on TNT with and without the transfer learning (pretraining). **e** The prediction performance on CL-20 with and without the transfer learning (pretraining). Green True Positive Rate (TPR), Orange True Negative Rate (TNR), Red Balanced Accuracy (BACC).

In order to validate generalization of CCGNet towards out-of-sample CCs (i.e., unseen CCs), we select three different types of co-crystals as independent test sets, which involve pharmaceutics, organic functional materials and energetic materials. For each type, we select some important cocrystal samples as representatives, which were reported to be challenging for the cocrystal screening. Figure 3a shows the number of positive and negative samples used in the three independent test sets. For achieving better prediction performance and stronger robustness, all the models adopt the ensemble learning strategy for these independent test sets, which combine the models from the tenfold cross-validation into an ensemble to “vote” on the prediction samples to obtain the final prediction result. As reported, the ensemble learning has been considered to be one of the most popular approaches for handling class imbalance^36^.

### Independent testing for pharmaceutical co-crystals

**Fig. 4:**
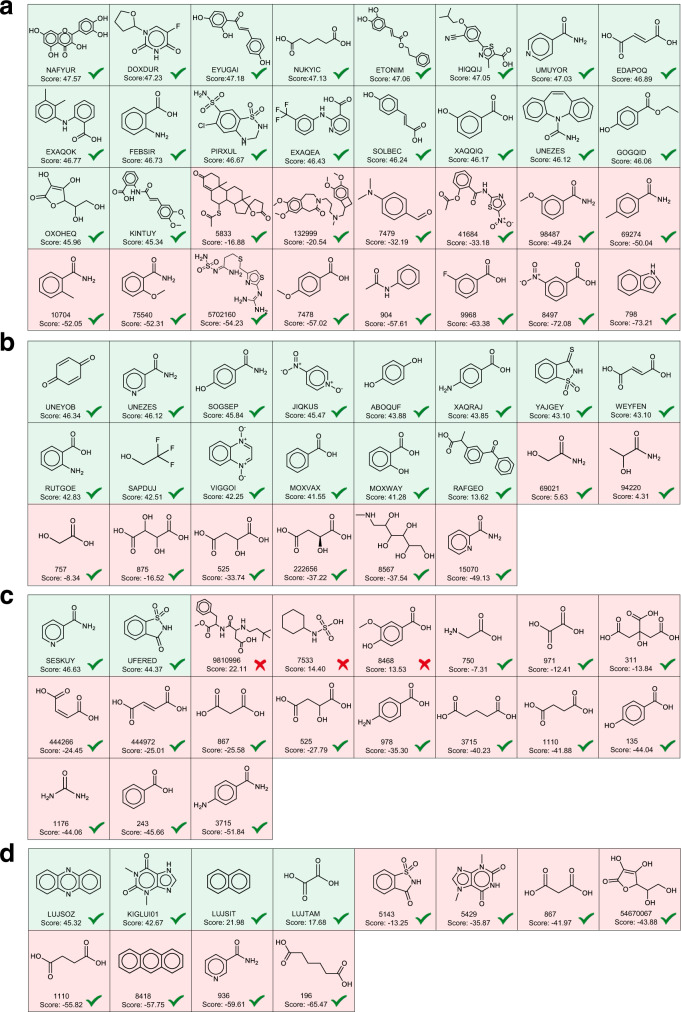
The predictive score ranking of CCGNet for the independent test sets of the four APIs. **a** Coformers of nicotinamide. **b** Coformers of carbamazepine. **c** Coformers of paracetamol. **d** Coformers of indomethacin. The scores are listed from high to low. The coformer of the positive sample is labeled as the CSD refcode while the negative sample is named in terms of PubChem Compound ID. Green background denotes true positive sample while red background represents true negative sample. The green and red ticks denote the correct prediction and the wrong prediction, respectively.

Pharmaceutical co-crystals can improve the physicochemical properties of potential APIs and simultaneously preserve their pharmacological properties, thus playing important roles in the pharmaceutical industry. In order to test the generalization ability of our model in the pharmacological co-crystals, we collect four APIs as study cases, which include Nicotinamide, Carbamazepine, Indomethacin, and Paracetamol. Nicotinamide is a harmless and widely used food additive^54,55^ and is often used as a coformer for co-crystallization^56–58^. Carbamazepine is used in the treatment of epilepsy and neuropathic pain. Due to limited bioavailability like low solubility, carbamazepine is generally needed to use a higher dose to achieve the desired therapeutic effect while co-crystallization is an effective method to improve its solubility^59–61^. In addition, the co-crystal prediction on Indomethacin and Paracetamol exhibited poor performance in previously developed approaches, which was considered to the lack of account for the crystallinity contribution to cocrystal formation by Sun et al.^17^. To address this issue, they developed two virtual coformer screening approaches based on a modern cloud-computing CSP technology at a dispersion-corrected density functional theory (DFT-D) level, which significantly improved the prediction performance with respect to the other methods like Hansen Solubility Parameter, COSMO-RS, and SSIPS^62–64^. However, the CSP method requires high computation cost, thus limiting its generalization in practice. In order to test whether our CCGNet model can achieve high accuracy, we also added the two challenging APIs (indomethacin and paracetamol) into the independent test sets (Fig. 3a). Supplementary Tables 5–8 show details regarding the positive and negative samples in the independent test set for the four APIs while Fig. 3b and Table 4 show the prediction performance on them. In addition, we sum the predictive score for the positive class over the CCGNet ensemble and sort them from high to low in Fig. 4 for visualization of the cocrystalization trend involving the four AIPs. In general, the higher the predictive score for the positive class, the greater the possibility of co-crystallization.

**Table 4:** Prediction performances of all the models on the co-crystals involving Nicotinamide (Nico), Carbamazepine (Carb), Indomethacin (Indo), and Paracetamol (Para).

Model	Metrics	Nico	Carb	Indo	Para	All APIs^a^
SVM	TPR (%)	100	92.86	100	100	97.3
	TNR (%)	71.43	50	64.71	75	65.96
	BACC (%)	85.71	71.43	82.35	87.5	81.63
RF	TPR (%)	100	100	100	100	98.95
	TNR (%)	100	37.5	52.94	100	75.47
	BACC (%)	100	68.75	76.47	100	86.17
DNN-des	TPR (%)	100	100	100	100	100
	TNR (%)	85.71	62.5	47.06	100	70.21
	BACC (%)	92.86	81.25	73.53	100	85.11
DNN-FP^30^	TPR (%)	94.44	92.86	100	25	89.19
	TNR (%)	42.86	37.5	88.24	75	63.83
	BACC (%)	68.65	65.18	94.12	50	76.51
enn-s2s^47^	TPR (%)	88.89	92.86	100	50	89.19
	TNR (%)	57.14	37.5	82.35	75	63.83
	BACC (%)	73.02	65.18	91.18	62.5	76.51
Graph-CNN^48^	TPR (%)	94.44	92.86	100	50	89.19
	TNR (%)	42.86	62.5	76.47	75	63.83
	BACC (%)	68.65	77.68	88.24	62.5	76.51
GCN^30^	TPR (%)	88.89	92.86	100	25	83.78
	TNR (%)	50	87.5	82.35	100	76.6
	BACC (%)	69.44	90.18	91.18	62.5	80.19
CCGNet	TPR (%)	100	100	100	100	100
	TNR (%)	100	100	82.35	100	93.62
	BACC (%)	100	100	91.18	100	96.81

^a^All APIs denote the holistic accuracy over Nico, Carb, Indo, and Para.

It can be seen from Fig. 3b and Table 4 that our CCGNet achieves 100% accuracy for three of the four APIs (Nicotinamide, Carbamazepine, and Paracetamol), where their positive samples and the negative ones are completely separated in the score rank (Fig. 4a–c). Although CCGNet does not get the highest accuracy for the indomethacin, it still achieves 91.18%, only inferior to DNN-FP^30^ (94.12%) and being ranked second. Despite the highest accuracy of DNN-FP on Indomethacin, it exhibits poor performance with lower than 70% on the other three APIs, much lower than our CCGNet, as shown by Table 4. In addition, although there are three prediction errors given by CCGNet for the negative samples of the indomethacin, the CCGNet model is still able to separate the three negative samples from the true positive samples in the score ranking (Fig. 4d), indicating that it still well evaluates the co-crystallization trend of the indomethacin. For RF, it also reaches 100% accuracy for Nicotinamide and Paracetamol, but its BACCs are only 68.75% and 76.47% for Carbamazepine and Indomethacin, respectively. For the other models including GCN^30^, their performances are also significantly inferior to CCGNet, in particular for the negative samples. In addition, it is worth noting that RF, SVM and DNN-des models only using the 12 molecular descriptors from the prior knowledge achieves the holistic BACC of 81.63–86.17% over the four APIs and higher than the four DL models only using the molecular graph or the molecular fingerprint (76.51–80.19%), further showcasing the importance of domain knowledge in the ML-based prediction. Overall, our CCGNet exhibits the highest holistic BACC (96.81%) over the four APIs and is greatly superior to the seven competitive models, further highlighting the advantages of our feature complementary and model framework. Compared to the CSP-based screening method with high computational cost^17^, CCGNet does not need to conduct the complex quantum mechanics calculation to obtain the information of the crystallinity but still achieves high prediction accuracy for these challenging APIs.

### Independent testing for π–π co-crystals

In the field of organic functional materials, cocrystal has become a promising approach to construct new functional materials^2,65^, ranging from photonic to optical and electronic materials. Polycyclic aromatic hydrocarbons (PAHs) with rich π-orbitals make electrons mobility through intermolecular π–π interaction, thus being promising components to form co-crystals that have diverse electrical and optical properties^66^. However, compared to strong interactions like H-bonding or halogen bonding, the π–π interaction is relatively weak, leading to a larger difficulty in cocrystal synthesis and structure determination. Thus, it is highly desired to accurately predict the π–π cocrystal system^9,67^. Pyrene is an important PAH. As a strong electron donor, it can be combined with a variety of materials to form an electron donor–acceptor system, which has been used in fluorescent probes, organic semiconductors, and optoelectronic materials^45,68^. Therefore, we select pyrene as a case to validate the generalization performance of CCGNet to the π–π CCs. As shown in Fig. 3a, the independent test set involving pyrene contains 58 positive samples and 6 negative ones collected from experiment reports (see Supplementary Table 9 for details). Figure 3c shows the prediction performances on Pyrene for our CCGNet and the seven competitive models. Excepting for GCN (89.94%), all the models achieve very high BACC (>98%). In particular, our CCGNet and SVM show 100% accuracy. As reflected by Supplementary Fig. 6, all the positive and negative samples involving pyrene are completely separated.

### Application and experimental validation for energetic co-crystals (ECCs)

Energetic materials (explosives, propellants, and fireworks) play important roles in military and civilian fields. However, the contradiction between the power and the sensitivity of explosives has been a well-known challenging problem, for example, the high-energy explosive generally exhibits low safety and vice versa^69,70^. The cocrystal engineering exhibits great potential in improving performance like stability, sensitivity, and oxygen balance^43^. However, the energetic molecules are often rich in nitro groups and lack of functional groups that devote important contribution to the traditional organic CCs, leading to larger difficulty in synthesis^5,10^. Therefore, a model that effectively predicts the formation of the energetic cocrystal will be an attractive tool for the experimental researches. Inspired by the issue, we apply CCGNet to the challenging task. Herein, we select two classic energetic explosives 2,4,6,8,10,12-hexanitrohexaazaisowurtzitane (CL-20) and 2,4,6-Trinitrotoluene (TNT) as independent cases. CL-20 is the most powerful non-nuclear energetic compound in practice^71^, yet its main disadvantage is its high sensitivity. The co-crystallization is an effective mean to improve its sensitivity. Compared to CL-20, TNT only has modest detonation velocity, but its advantage is low sensitivity to the impact^72^. Similarly, the co-crystallization between TNT and other explosives with high sensitivity could improve the comprehensive performance (high-energy and low sensitivity). Thus, we collected the 41 positive samples and 14 negative ones involving TNT and CL-20 as the independent test set (Fig. 3a).

**Fig. 5:**
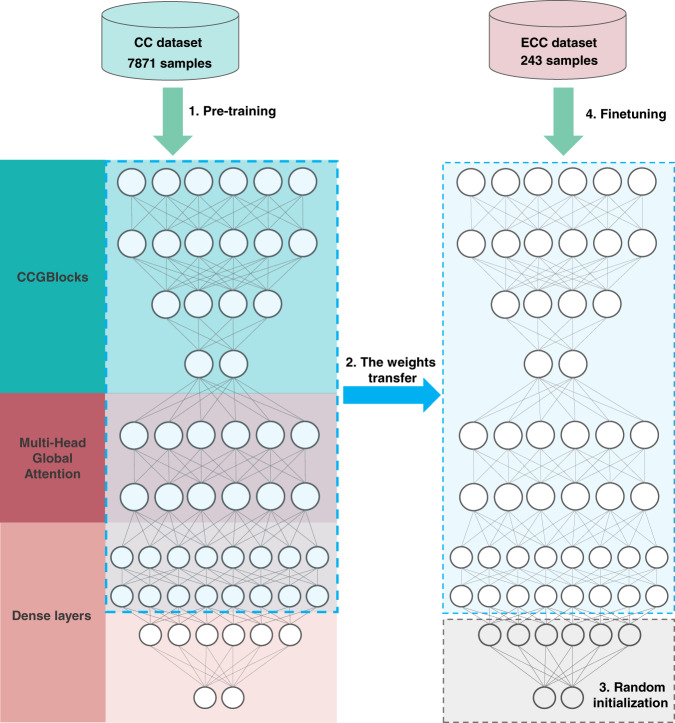
The flow chart of the transfer learning for the energetic co-crystals. The energetic cocrystal prediction model is also based on the CCGNet framework involving CCGBlocks, Multi-head Global attention and dense layers. The CC dataset is first applied to pretrain the model. Then the weights pretrained on CC dataset are served as initialization weights of CCGBlocks, Multi-Head Global Attention and part of dense layers (boxes surrounded by blue dotted-lines), which is called as weight transfer. Then, the last two dense layers are initialized randomly (Gray box). Finally, ECC dataset is used to finetune all the weights of the model.

However, when we directly apply the CCGNet model and the seven competitive ones trained on the cocrystal dataset (i.e., CC dataset) containing 7871 samples to the independent test set of TNT and CL -20, the balanced accuracies are very low, lower than 61% for TNT and 59% for CL-20 (see Fig. 3d, e and Supplementary Table 10), different from the high performance on the pharmaceutical and π–π CCs. The reason should be attributed to the fact that the energetic molecules have significantly different structures from common organic CCs from CSD, for example, rich nitro groups or caged structures like CL-20. Thus, the knowledge learned by the ML models on the cocrystal dataset is lack of the unique structural information, leading to the poor performance. To cape with the problem, it is necessary to effectively integrate the knowledge from the very limited energetic CCs into our CCGNet model trained on the large amount of the traditional CCs. Therefore, we adopt the transfer learning strategy, as illustrated by Fig. 5.

Concretely, we use the weights of pretrained models on the CC dataset as initialization weights of CCGBlocks, Multi-Head Global Attention and part of dense layers while only the last two dense layers are initialized randomly (Fig. 5). In order to finetune the model weights, we need to construct an additional energetic cocrystal dataset (called ECC dataset below) to highlight the knowledge from the energetic CCs. To the end, we collect 116 ECC positive samples from CSD, as shown in Supplementary Table 11. Unfortunately, there are no public reports on failed experiments on the energetic cocrystal, leading to the difficulty in obtaining the negative samples. Herein, we combine the experimental experiences and the ISPE method proposed by Musumeci et al.^15^ to construct the negative sample set. Supplementary Fig. 7 shows representative coformers used to construct the energetic cocrystal negative samples. Supplementary Table 12 shows the calculated results from the ISPE method for 864 co-crystal combination pairs. Finally, 127 pairs are selected as the negative samples for the energetic co-crystals. Detailed descriptions regarding the construction of the negative samples are presented in Supplementary Methods. Consequently, the ECC dataset applied to finetune the CCGNet model contains 116 positive samples and 127 negative ones.

**Fig. 6:**
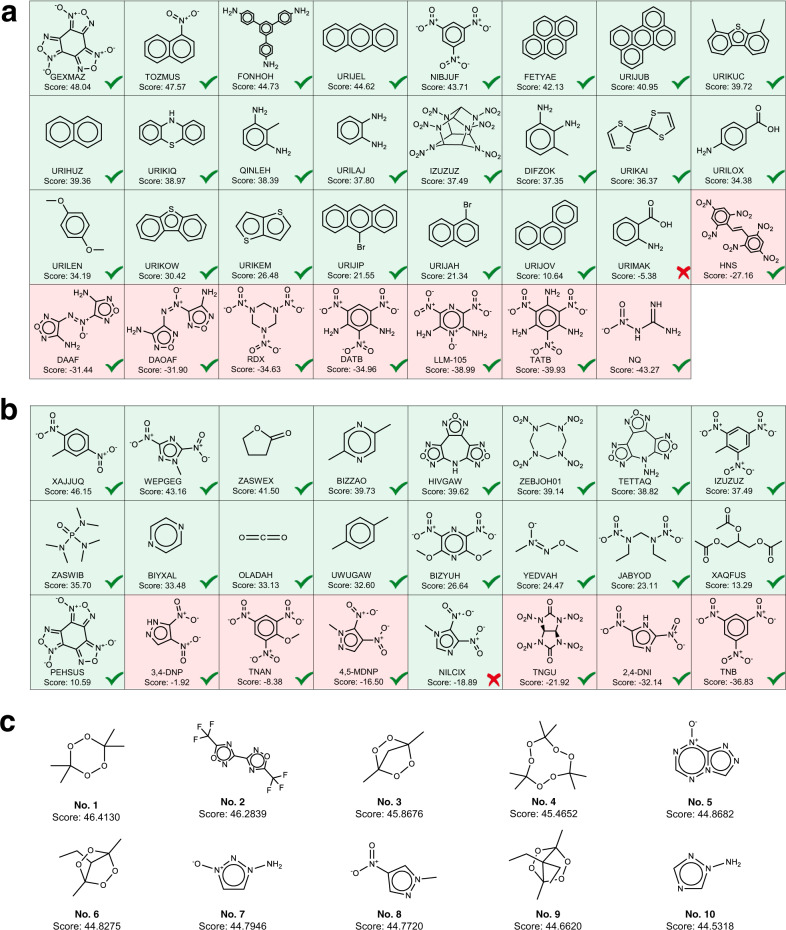
Score ranking predicted for the independent testing set of the energetic cocrystals. **a** Coformers of TNT. **b** Coformers of CL-20. **c** Structures of top ten coformers screened for CL-20. The green and red backgrounds indicate the true positive sample and true negative sample, respectively. The green tick denotes the correct prediction of the model while the red cross represents the wrong prediction. The score higher, the more likely to crystallize with CL-20.

We use the 243 energetic cocrystal samples to finetune the 10 pretrained models derived from the CC dataset and each model is subjected to 5-fold random cross-validation to obtain 50 energetic cocrystal predictive models. Then, ten models with the lowest loss in the validation set (Supplementary Table 13) are selected as the ensemble to predict cocrystal formation of TNT and CL-20, respectively. Figure 3d, e shows the performance on the independent test set after finetuning. It is clear that the predictive performance is remarkedly improved by the transfer learning. The ensembled BACCs are improved to be 97.83% for TNT and 97.22% for CL-20. Despite one wrong prediction observed for TNT, the positive and negative samples are still completely separated by the score ranking for TNT, as evidenced by Fig. 6a. For CL-20, seventeen of the total 18 positive samples are exactly the top-ranked hits in the scoring list of CL-20 and only one positive sample is low-ranked so that mixed with the negative samples and wrongly predicted, as reflected by Fig. 6b. Overall, our model almost captures the co-crystallization trends for TNT and CL-20, which can be served as a virtual screening tool to provide guidelines for the subsequent experiments.

To gauge the reliability of our model in practical application on one side, and explore new cocrystal for CL-20 on the other side, we collect 435 potential energetic compounds reported and used the finetuning CCGNet model to screen new potential coformers for CL-20. Figure 6c lists ten coformers screened in the top 10 ranked hits for CL-20, including five peroxides (coformer **1**, **3**, **4**, **6**, and **9**), one fluorides (coformer **2**), one triazole derivatives (coformer **10**) and three energetic molecules only containing C, H, O, and N atoms (coformers **5**, **7**, **8**). Considering usually high sensitivity of the peroxides^73,74^ and the requirement to environmental safety for modern explosives^75,76^. we first exclude the six coformers involving the peroxides and fluorides (cofomers **1**, **2**, **3**, **4**, **6**, and **9**) in the subsequent experiment on co-crystalization with CL-20. For the remaining coformers **5**, **7**, **8** and **10**, we calculate their impact sensitivities and explosion heats that are two important properties involving the safety and the explosion performance. The calculation methods are described in Supplementary Methods and the calculated results are listed in Supplementary Table 15. Trading off the impact sensitivity and the explosion heat, we finally select the coformer **8** (1-methyl 4-nitropyrazole) to conduct the cocrystalization experiment (see Supplementary Methods for details). We mingle the coformer **8** and CL-20 with anhydrous methanol. After slowly evaporating the anhydrous methanol solution, the crystal is obtained, which is further analyzed by single crystal X-ray diffraction. Crystallographic data (Supplementary Table 16 and Supplementary Fig. 8) proves that a new CL-20/1-methyl-4-nitropyrazole cocrystal (CSD deposition number: 2107286) is formed. More experimental details are described in Supplementary Methods.

## Discussion

Here we develop a GNN-based DL model coupled with the feature complementary strategy to accurately predict the formation of the cocrystal. A reliable cocrystal dataset is obtained by collecting 1052 negative samples from the experimental literature to minimize the false negative, along with 6819 positive samples from CSD. The model is strongly validated by seven competitive models including the traditional ML and the classical graph neural network (GNN) reported, supplemented by the three different and challenging out-of-sample tests (pharmaceutical CCs, π–π CCs, and energetic CCs). Benefited from the complementary feature representation and the flexible GNN-based framework, our model greatly outperforms the seven competitive models in the imbalanced dataset. Crucially, CCGNet achieves high prediction accuracy with >96% for the diverse data from different cocrystal spaces as unseen cases, exhibiting strong robustness and generalization. Finally, the experimental validation on a new energetic-energetic cocrystal of CL-20/1-methyl-4-nitropyrazole predicted further confirms the reliability of our model and high potential in practice. The result clearly confirms that embedding important priori knowledge can improve the performance of the DL, in particular for the limited dataset available. Collectively, these important technical advantages presented by our work, including the data augmentation, the feature representation and the flexible model architecture coupled with the attention mechanism and the transfer learning, could provide helpful guidelines for the application of the DL in practice. We also integrated the ensemble model as a pipeline that can provide the high throughput screening for the defined compounds pairs and generate a report form automatically. All Source Codes and Data are freely available at https://github.com/Saoge123/ccgnet. We expect that they will become a useful tool for aiding the design of cocrystal materials.

## Methods

### Node update function ${{{{{{\mathbf{\Phi }}}}}}}_{{{{{{\bf{v}}}}}}}$

Graph-CNN, a spatial-based graph convolution network from Such et al.^48^ is used for the message passing and node update. The Graph-CNN relies on convolutional filter H to propagate and update node features. **H** is a *N* × *N* × *C* filter tensor, which is a stack of *N* × *N* filter matrices indexed by the node feature they filter. *N* is node number and *C* is the number of node feature. ${{{{{{\bf{H}}}}}}}^{({{{{{\boldsymbol{c}}}}}})}\,$ is defined in terms of Eq. (1):

1 $$\begin{array}{c}{{{{{{\bf{H}}}}}}}^{\left(c\right)}\,=\,\mathop{\sum }\limits_{l\,=\,1}^{L}{{h}_{l}^{\left(c\right)}{{{{{\bf{A}}}}}}}_{l}\end{array}$$

${{{{{{\bf{A}}}}}}}_{l}$ is the *l*-th slice of adjacency tensor$\,{{{{\boldsymbol{{{{{\mathscr{A}}}}}}}}}}$ whose shape is *N* × *N* × *L*. ${h}_{l}^{{{{{{\boldsymbol{(}}}}}}c{{{{{\boldsymbol{)}}}}}}}$ is a scalar corresponding to a given input feature and a given slice of ${{{{{{\bf{A}}}}}}}_{l}$. *L* is the number of edge feature. The operation that filters the node feature ${{{{{{\bf{V}}}}}}}_{{{{{{\rm{in}}}}}}}$ is defined by Eq. (2)

2 $$\begin{array}{c}{{{{{{\bf{V}}}}}}}_{{{{{{\rm{out}}}}}}}\,=\,\mathop{\sum }\limits_{c\,=\,1}^{C}{{{{{{\bf{H}}}}}}}^{\left(c\right)}{{{{{{\bf{V}}}}}}}_{{{{{{\rm{in}}}}}}}^{\left(c\right)}\,+\,b\end{array}$$

where ${{{{{{\bf{V}}}}}}}_{{{{{{\rm{in}}}}}}}^{(c)}\,\in \,{{\mathbb{R}}}^{N\,\times\, 1}$ represents the *c*-th node feature that is the column of ${{{{{{\bf{V}}}}}}}_{{{{{{\rm{in}}}}}}}$. *b* is a scalar and ${{{{{{\bf{V}}}}}}}_{{{{{{\rm{out}}}}}}}\,\in \,{{\mathbb{R}}}^{N\,\times\, 1}$ is the result of the operation that filter the node feature ${{{{{{\bf{V}}}}}}}_{{{{{{\rm{in}}}}}}}$.

Here, multiple filters can be set by adding another dimension to ${{{{{\bf{H}}}}}}$ and then it becomes a tensor $\,\in \,{{\mathbb{R}}}^{N\,\times\, N\,\times\, C\,\times\, F}$. As a result, the output ${{{{{{\bf{V}}}}}}}_{{{{{{\rm{out}}}}}}}$ (Eqs. (3, 4) also becomes a tensor $\in {{\mathbb{R}}}^{N\,\times\, F}$.

3 $$\begin{array}{c}{{{{{{\bf{V}}}}}}}_{{{{{{\rm{out}}}}}}}^{\left(f\right)}\,=\,\mathop{\sum }\limits_{c\,=\,1}^{C}{{{{{{\bf{H}}}}}}}^{\left(c,f\right)}{{{{{{\bf{V}}}}}}}_{{{{{{\rm{in}}}}}}}^{\left(c\right)}\,+\,b\end{array}$$

4 $$\begin{array}{c}{{{{{{\bf{V}}}}}}}_{{{{{{\rm{out}}}}}}}\,=\,\,{\parallel }_{f\,=\,1}^{F}{{{{{{\bf{V}}}}}}}_{{{{{{\rm{out}}}}}}}^{\left(f\right)}\end{array}$$

where ${{{{{{\bf{V}}}}}}}_{{{{{{\rm{out}}}}}}}^{(f)}$ is a column of ${{{{{{\bf{V}}}}}}}_{{{{{{\rm{out}}}}}}}\,\in \,{{\mathbb{R}}}^{N\,\times\, F}$ and $\parallel$ is concatenation. For brevity, this operation is also written as Eq. (5)

5 $$\begin{array}{c}{{{{{{\bf{V}}}}}}}_{{{{{{\rm{out}}}}}}}={{{{{\rm{GConv}}}}}}\left({{{{{{\bf{V}}}}}}}_{{{{{{\rm{in}}}}}}},F\right)+{{{{{\bf{b}}}}}}\end{array}$$

Finally, to consider self-loop of nodes and activation function, the convolutional operation can be described as Eq. (6)

6 $$\begin{array}{c}{{{{{{\bf{V}}}}}}}_{{{{{{\rm{out}}}}}}}\,=\,{{{{{\rm{\sigma }}}}}}\left({{{{{\bf{I}}}}}}{{{{{{\bf{V}}}}}}}_{{{{{{\rm{in}}}}}}}{{{{{{\bf{W}}}}}}}_{0}\,{{{{{\boldsymbol{+}}}}}}\,{{{{{\rm{GConv}}}}}}\left({{{{{{\bf{V}}}}}}}_{{{{{{\rm{in}}}}}}},F\right)\,+\,{{{{{\bf{b}}}}}}\right)\end{array}$$

${{{{{\rm{\sigma }}}}}}$ is activation function (ReLU^77^ used in this work). ${{{{{\bf{I}}}}}}$ is a diagonal matrix that represents self-loop of nodes. Here ${{{{{{\bf{W}}}}}}}_{0}$ is trainable weight and ${{{{{\bf{b}}}}}}\,\in\, {{\mathbb{R}}}^{F}$ is bias.

### Global state function ${{{{{{\mathbf{\Phi }}}}}}}_{{{{{{\bf{u}}}}}}}$

A single-layer feedforward neural network is used as global state function to perform nonlinear transformation for the global attribute of molecules. It is defined by Eq. (7):

7 $$\begin{array}{c}{{{{{{\boldsymbol{u}}}}}}}_{{{{{{\boldsymbol{out}}}}}}}\,=\,{{{{{\boldsymbol{\sigma }}}}}}\left({{{{{\boldsymbol{uW}}}}}}\,+\,{{{{{\boldsymbol{b}}}}}}\right)\end{array}$$

where ${{{{{\bf{u}}}}}}$ is the global attribute of a molecule; ${{{{{\rm{\sigma }}}}}}$ is activation function (ReLU^77^ in this work). ${{{{{\bf{W}}}}}}$ and ${{{{{\bf{b}}}}}}$ are trainable weight and bias, respectively.

### Concatenation operation ${{{{{{\rm{\rho }}}}}}}^{{{{{{\bf{u}}}}}}\to {{{{{\bf{v}}}}}}}$

In CCGBlock, ${{{{{{\rm{\rho }}}}}}}^{{{{{{\bf{u}}}}}}{{{{{\boldsymbol{\to }}}}}}{{{{{\bf{v}}}}}}}$ concatenates the global state (i.e., the 12 molecular descriptors) of each co-former and the node embeddings together. Cocrystal input (CCGraph) can be expressed as Eq. (8):

8 $$\begin{array}{c}{{{{{\rm{CCGraph}}}}}}\,=\,\left\{{{{{{\bf{U}}}}}}\left({{{{{{\bf{u}}}}}}}_{1},{{{{{{\bf{u}}}}}}}_{2}\right),{{{{{\bf{A}}}}}}\left({{{{{{\bf{A}}}}}}}_{1},{{{{{{\bf{A}}}}}}}_{2}\right),{{{{{\bf{V}}}}}}\left({{{{{{\bf{V}}}}}}}_{1},{{{{{{\bf{V}}}}}}}_{2}\right)\right\}\end{array}$$

where the subscript refers to each co-former. ${{{{{{\bf{V}}}}}}}_{1}$and ${{{{{{\bf{V}}}}}}}_{2}$ can be expressed as Eqs. (9–10):

9 $$\begin{array}{c}{{{{{{\bf{V}}}}}}}_{{{{{{\bf{1}}}}}}}\,=\,\left({{{{{{\bf{v}}}}}}}_{1}^{1},{{{{{{\bf{v}}}}}}}_{1}^{2},\ldots ,{{{{{{\bf{v}}}}}}}_{1}^{i},\ldots ,{{{{{{\bf{v}}}}}}}_{1}^{n}\right)\end{array}$$

10 $$\begin{array}{c}{{{{{{\bf{V}}}}}}}_{{{{{{\bf{2}}}}}}}\,=\,({{{{{{\bf{v}}}}}}}_{2}^{1},{{{{{{\bf{v}}}}}}}_{2}^{2},\ldots ,{{{{{{\bf{v}}}}}}}_{2}^{j},\ldots ,{{{{{{\bf{v}}}}}}}_{2}^{m})\end{array}$$

where the subscript refers to each co-former and the superscript denotes each atom. We perform the concatenation for every atom in terms of Eqs. (11–12):

11 $$\begin{array}{c}{{{{{{{\bf{v}}}}}}}_{1}^{i}}^{{\prime} }\,=\,{{{{{{\bf{v}}}}}}}_{1}^{i}\,\bigoplus\, {{{{{{{\bf{u}}}}}}}_{{{{{{\bf{1}}}}}}}}^{{\prime} }\end{array}$$

12 $$\begin{array}{c}{{{{{{{\bf{v}}}}}}}_{2}^{j}}^{{\prime} }\,=\,{{{{{{\bf{v}}}}}}}_{2}^{j}\,\bigoplus\, {{{{{{{\bf{u}}}}}}}_{{{{{{\bf{2}}}}}}}}^{{\prime} }\end{array}$$

where ${{\bigoplus }}$ denotes concatenation operation.

### Readout function

Herein, we use multi-head global attention as the readout function. Following the way of human thinking, the attention mechanism uses limited attention resources to quickly screen out high-value information from a large amount of information, which has achieved remarkable performance in different tasks, for example, natural language processing^78^, image classification^79^ and speech recognition^80^. Thus, we introduce the attention mechanism in the readout function to further optimize the feature space derived from the message passing phase. Through highlighting atoms by the attention weights, we can explore how model learns the chemical structure and make the model interpretable.

Global attention calculates the attention coefficient of each node based on node features. Then the feature at the graph level is obtained by summing the product of attention coefficient and corresponding node feature, as described by Eqs. (13, 14):

13 $$\begin{array}{c}{{{{{\bf{a}}}}}}\,=\,{{{{{\rm{softmax}}}}}}\left({{{{{\rm{\varphi }}}}}}\left({{{{{{\bf{X}}}}}}}_{{{{{{\rm{in}}}}}}}\right)\right)\end{array}$$

14 $$\begin{array}{c}{{{{{{\bf{X}}}}}}}_{{{{{{\rm{graph}}}}}}}\,=\,\mathop{\sum }\limits_{i\,=\,1}^{N}{a}_{i}{{{{{{\bf{x}}}}}}}_{i}\end{array}$$

where $\varphi$ denote neural network (MLP in this work), ${{{{{\bf{a}}}}}}\in {{\mathbb{R}}}^{N}$ is N-dimensional vector composed by attention coefficient of each node. ${{{{{{\bf{x}}}}}}}_{i}$ represents the feature of node *i*, which is a row of node features ${{{{{{\bf{X}}}}}}}_{{{{{{\rm{in}}}}}}}$.

Herein, we construct the multi-head attention into the global attention, which computes *K* attention coefficients of each node in parallel, yielding an attention matrix ${{{{{\boldsymbol{\alpha }}}}}}\in {{\mathbb{R}}}^{N\times K}$ (Eq. (15). Multi-head attention allows the model to jointly attend to information from different representation subspaces at different positions^78^.

15 $${{{{{\boldsymbol{\alpha }}}}}}={{{{{\rm{softmax}}}}}}\left({{\phi }}\left({{{{{{\bf{X}}}}}}}_{{{{{{\rm{in}}}}}}}\right)\right)$$

where ${{\phi }}$ denotes neural network (MLP in this work). Similar to the global attention, we calculate graph level embedding *K* times. As expressed by Eqs. (16, 17), these embeddings are concatenated to produce the final graph embedding ${{{{{{\bf{X}}}}}}}_{{{{{{\rm{graph}}}}}}}^{{{{{{\rm{cat}}}}}}}$ that is a $K\,\times\, C$ dimension vector.

16 $$\begin{array}{c}{{{{{{\bf{X}}}}}}}_{{{{{{\rm{graph}}}}}}}^{j}\,=\,\mathop{\sum }\limits_{i\,=\,1}^{N}{\alpha }_{i,j}{{{{{{\bf{x}}}}}}}_{i}\end{array}$$

17 $$\begin{array}{c}{{{{{{\bf{X}}}}}}}_{{{{{{\rm{graph}}}}}}}^{{{{{{\rm{cat}}}}}}}\,=\,{\,}_{j\,=\,1}^{\quad \!\! K}\Vert{{{{{\bf{X}}}}}}_{{{{{{\rm{graph}}}}}}}^{j}\end{array}$$

where ${{{{{{\bf{X}}}}}}}_{{{{{{\rm{graph}}}}}}}^{j}$ is graph embedding calculated by using the *j*-th version of attention coefficients that is the *j*-th column of ${{{{{\boldsymbol{\alpha }}}}}}$. *K* is the head number and ${\alpha }_{i,j}$ is an element of ${{{{{\boldsymbol{\alpha }}}}}}$.

### Training and metrics

In order to improve the robustness of ML, all the models including the seven competitive ones are trained on the data augmented by exchanging the permutation of a pair of coformers for the 10-fold cross-validation set. To avoid deceitful performance caused by the majority class prediction in the imbalance distribution of the positive and negative samples (6.5:1 ratio in the work), we use True Negative Rate (TNR) and True Positive Rate (TPR) to directly measure the classification performance on the positive and negative classes independently, through which Balanced Accuracy (BACC) can be obtained as an overall metric to trade off the accuracies between the positive samples and the negative ones, as defined by Eqs. (18, 20).

18 $$\begin{array}{c}{{{{{\rm{TPR}}}}}}\,=\,{{{{{\rm{Sensitivity}}}}}}\,=\,\frac{{{{{{\rm{TP}}}}}}}{{{{{{\rm{TP}}}}}}\,+\,{{{{{\rm{FN}}}}}}}\end{array}$$

19 $$\begin{array}{c}{{{{{\rm{TNR}}}}}}\,=\,{{{{{\rm{Specificity}}}}}}\,=\,\frac{{{{{{\rm{TN}}}}}}}{{{{{{\rm{FP}}}}}}\,+\,{{{{{\rm{TN}}}}}}}\end{array}$$

20 $$\begin{array}{c}{{{{{\rm{BACC}}}}}}\,=\,\frac{{{{{{\rm{TPR}}}}}}\,+\,{{{{{\rm{TNR}}}}}}}{2}\end{array}$$

where TP is True Positive; FP is False Positive; TN is True Negative; FN is False Negative. All models are trained with Adam^81^ optimizer.

### Model implementation

CCGNet is constructed under the opensource ML framework of TensorFlow^82^. CCGNet outputs two-dimensional vectors [a, b] which represent the predictive scores for negative and positive class, respectively. If b > a, the output is labeled as the positive sample, and vice versa. Supplementary Methods describes details regarding constructions of SVM, RF, DNN-des, DNN-FP, GCN, Graph-CNN, and enn-s2s. Bayesian optimization is used to search the optimal hyper-parameters for all the models (see Supplementary Methods). The representation of the samples is implemented by RDkit, OpenBabel, and CCDC Python Application Programming Interface. We train the models on Nvidia RTX 2080ti GPU.

## Supplementary information

Supplementary Information

Description of Additional Supplementary Files

Supplementary Data 1

Supplementary Data 2

## Data Availability

The positive and negative samples generated in this study are provided in the Supplementary Data 1, 2. The X-ray crystallographic coordinates for structure reported in this study have been deposited at the Cambridge Crystallographic Data Center (CCDC), under deposition number: 2107286. These data can be obtained free of charge from The Cambridge Crystallographic Data Center via www.ccdc.cam.ac.uk/data_request/cif.

## Code availability

To ensure reproducibility of the results, the source code of all the models used in this work can be acquired at https://github.com/Saoge123/ccgnet (10.5281/zenodo.5496365).
