# Supplementary material for: Coupling complementary strategy to flexible graph neural network for quick discovery of coformer in diverse co-crystal materials
Source: Nat Commun. 2021 Oct 12;12:5950. doi: 10.1038/s41467-021-26226-7 (PMC8511140; doi:10.1038/s41467-021-26226-7)
Supplement: Supplementary file 1 — Supplementary Information [file 41467_2021_26226_MOESM1_ESM.pdf]

Supplementary Information

Coupling Complementary Strategy to Flexible Graph Neural Network for Quick Discovery of Coformer in Diverse Co-crystal Materials

Y. Jiang et al.

Supplementary Figures

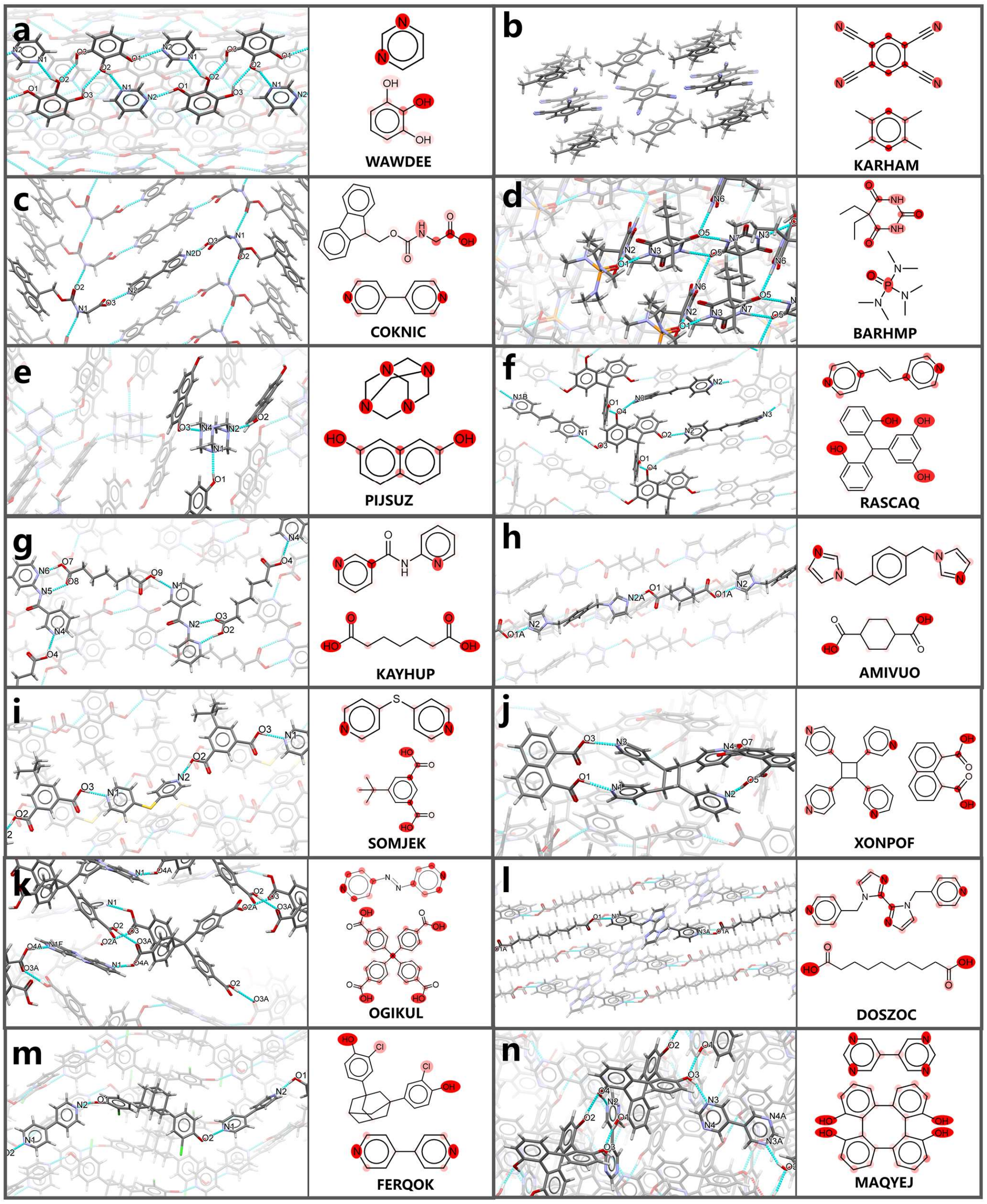

Supplementary Figure 1: Attention visualization for some representative cocrystals. **a** WAWDEE. **B** KARHAM. **c** COKNIC. **d** BARHMP. **e** PIJSUZ. **f** RASCAQ. **g** KAYHUP. **h** AMIVUO. **i** SOMJEK. **j** XONPOF. **k** OGIKUL. **l** DOSZOC. **m** FERQOK. **n** MAQYEJ. The real cocrystal structure is displayed by Mercury while the corresponding 2D structure is highlighted by the attention weights derived from CCGNet. The redder the color, the greater the attention weight. The cyan dash line denotes the intermolecular H-bonding.

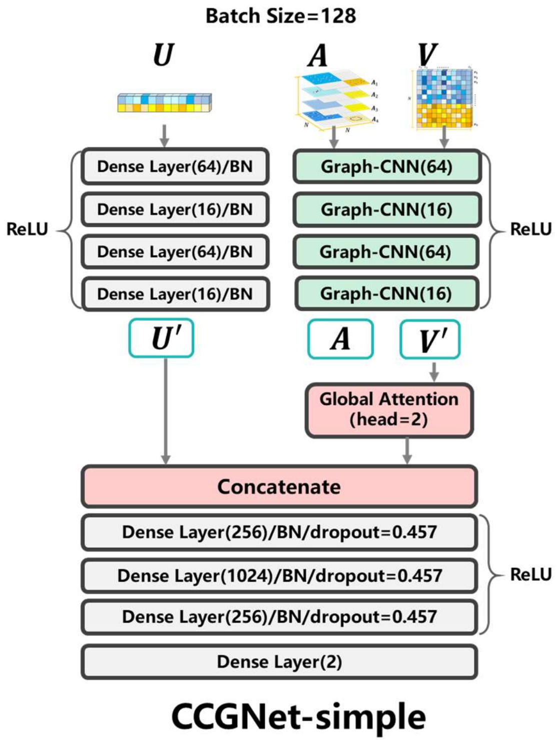

Supplementary Figure 2: Configuration of CCGNet-simple.

The concatenation operation between the node feature and the global state in each CCGBlock is removed, only retaining the concatenation at the readout stage.

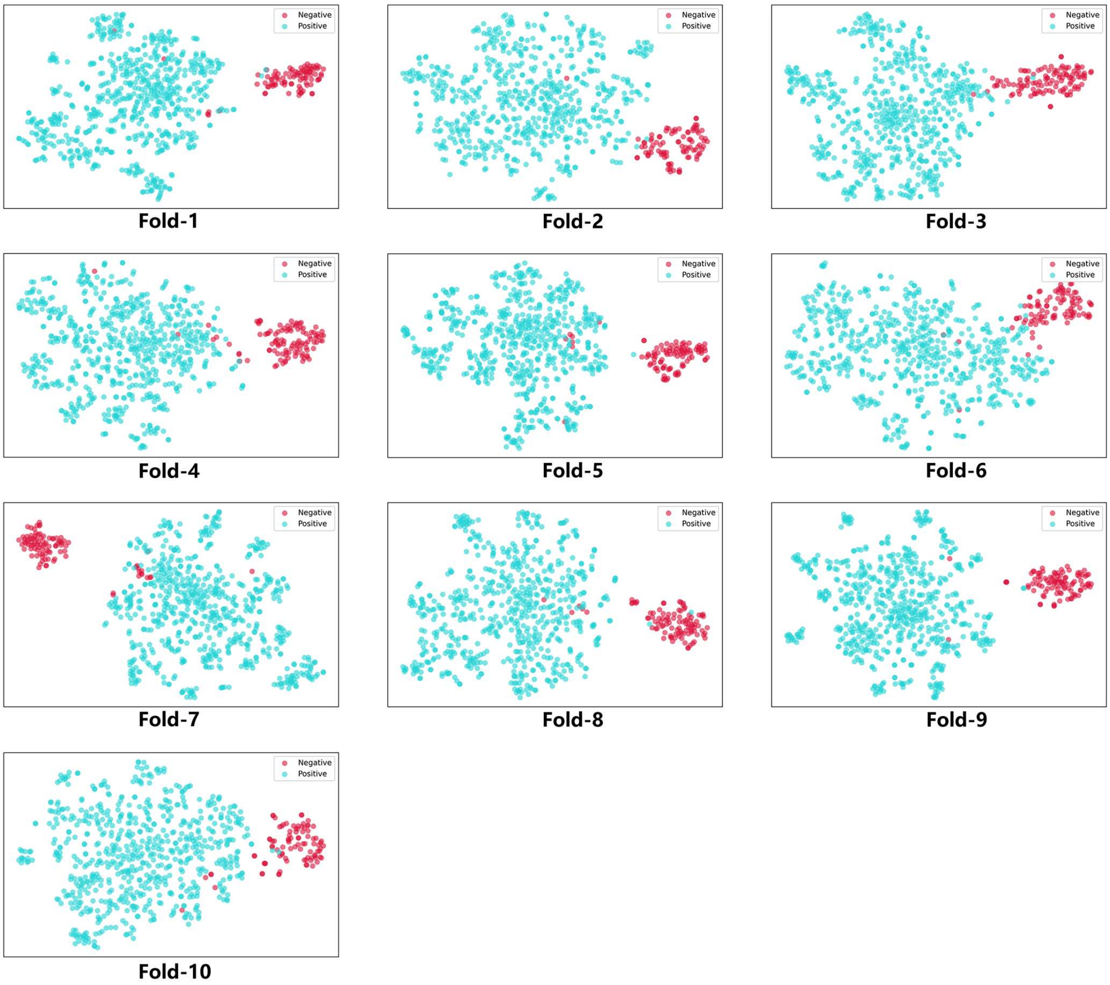

Supplementary Figure 3: t-SNE analysis on each fold of the 10-fold cross-validation for CCGNet.

Hidden representations are extracted after the concatenation operation of the readout phase. Blue dot: positive sample. Red dot: negative sample.

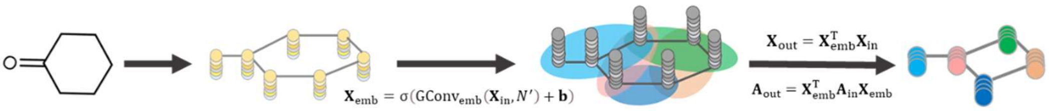

Supplementary Figure 4: The process of Graph Embed Pooling.

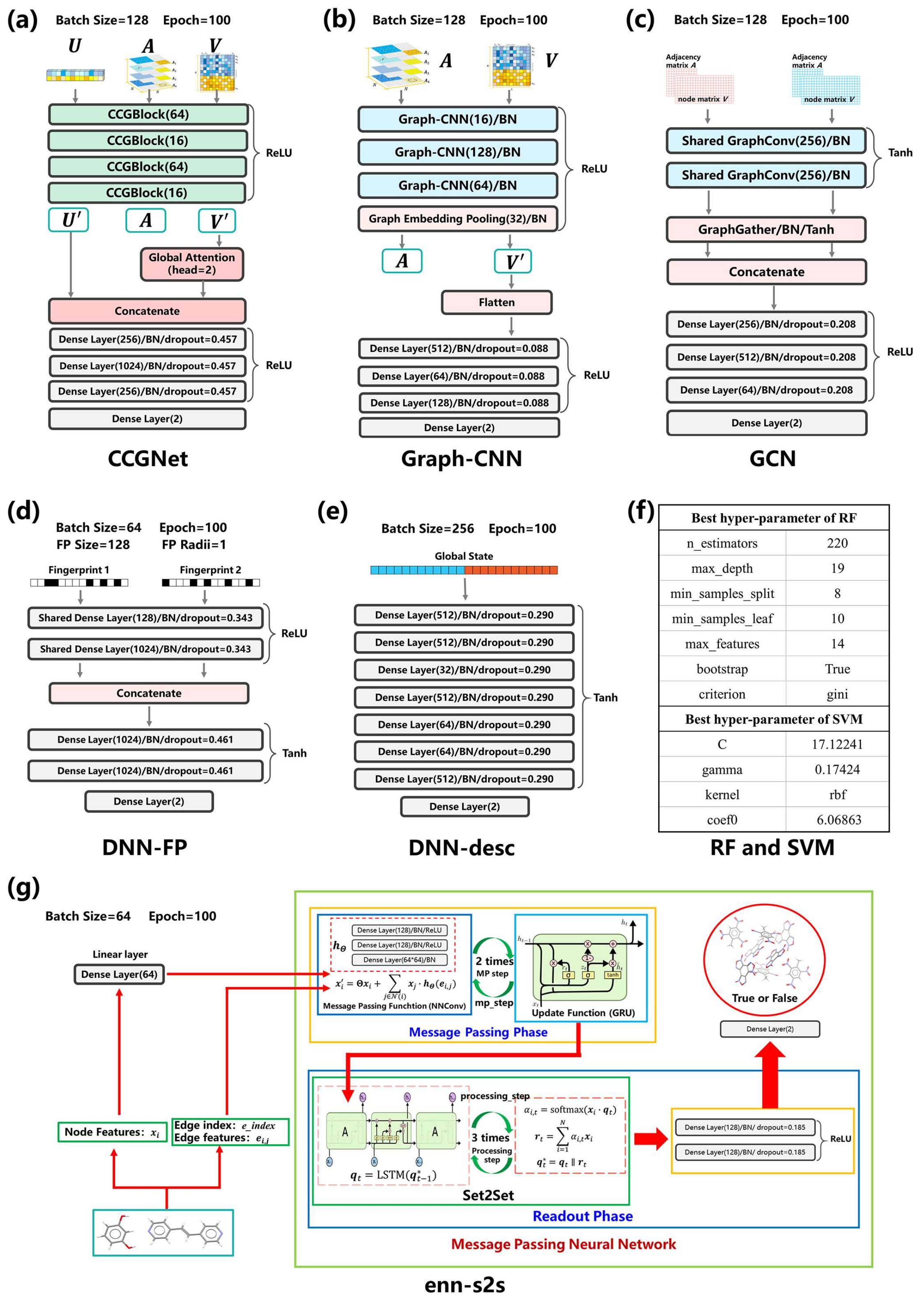

Best hyper-parameter of RF	
n_estimators	220
max_depth	19
min_samples_split	8
min_samples_leaf	10
max_features	14
bootstrap	True
criterion	gini
**Best hyper-parameter of SVM**	
C	17.12241
gamma	0.17424
kernel	rbf
coef0	6.06863

Supplementary Figure 5: The best configurations of all the models, derived from the optimized hyper-parameters.

(a) CCGNet. **(b)** Graph-CNN. **(c)** GCN. **(d)** DNN-FP. **(e)** DNN-desc. **(f)** RF and SVM. **(g)** enn-s2s.

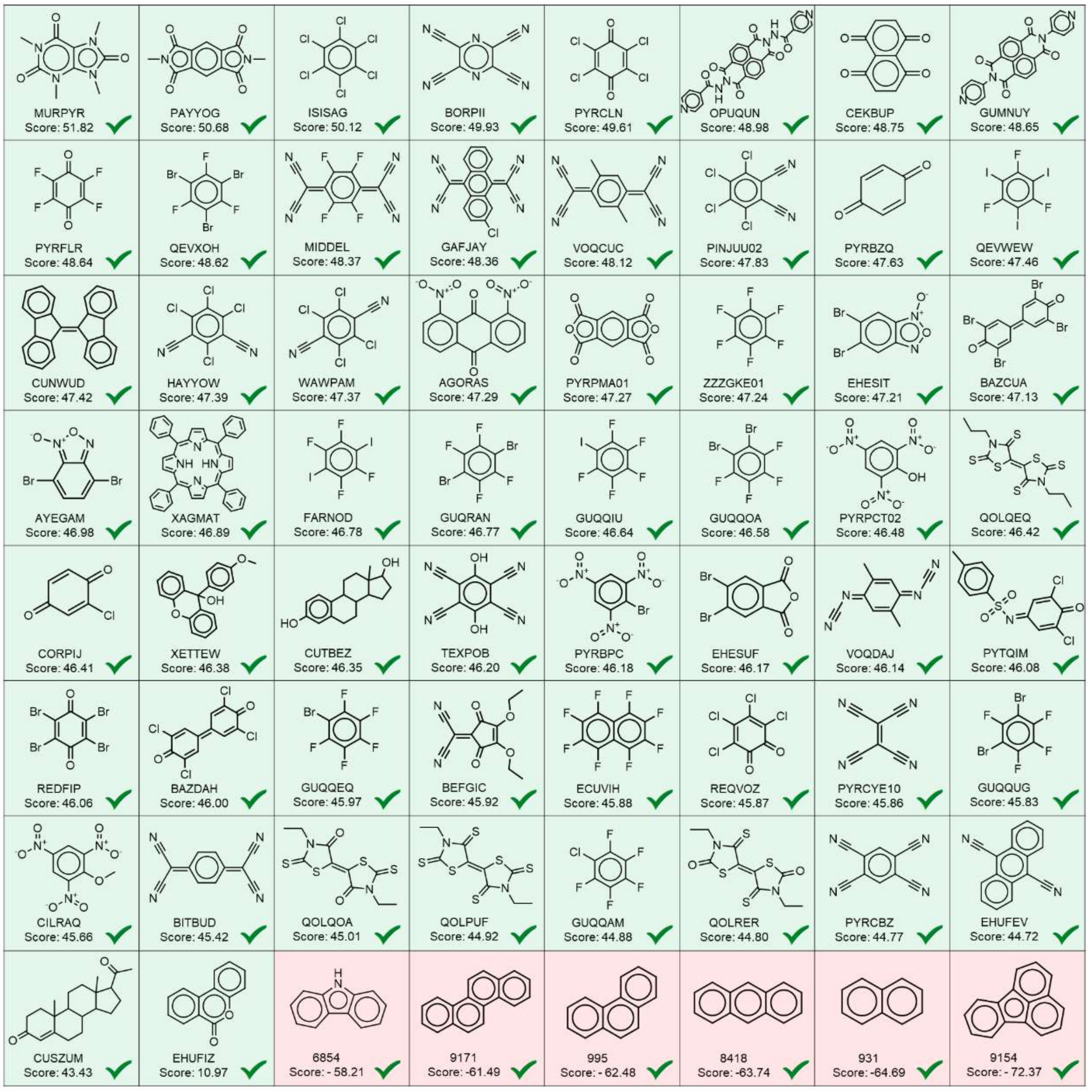

Supplementary Figure 6: The predictive score ranking of CCGNet from high to low for the coformers of Pyrene.

Green background: True Positive sample; red background: True Negative sample. The green tick and red cross denote the correct prediction and the wrong prediction, respectively. The coformer of the positive sample is labeled as the CSD refcode while the negative sample is named in terms of PubChem Compound ID.

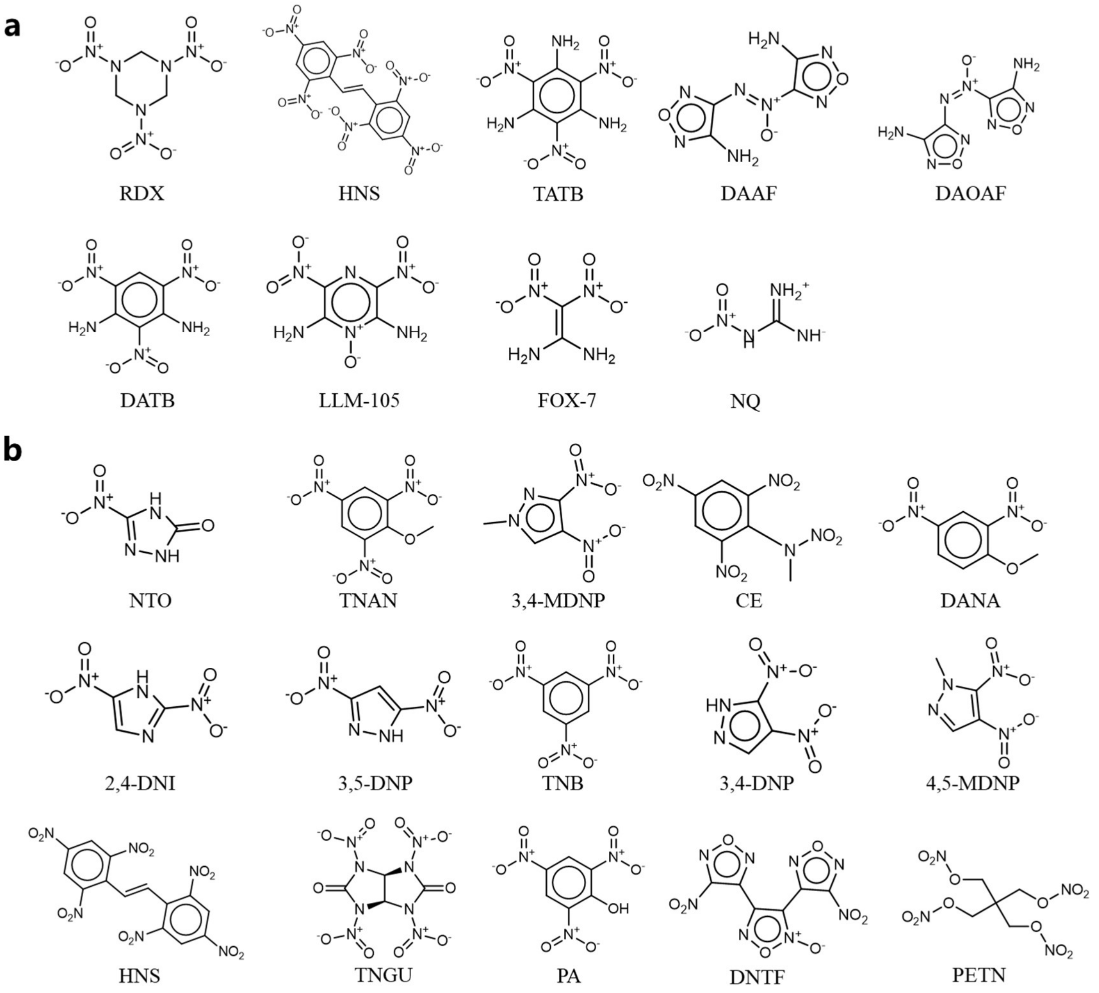

Supplementary Figure 7: Molecules used to construct the energetic cocrystal negative samples.

a Structures of nine energetic molecules served as coformers of potential negative samples for the ISPE calculation. **b** Structures of fifteen coformers derived from failed co-crystallization with CL-20 in our experimental works.

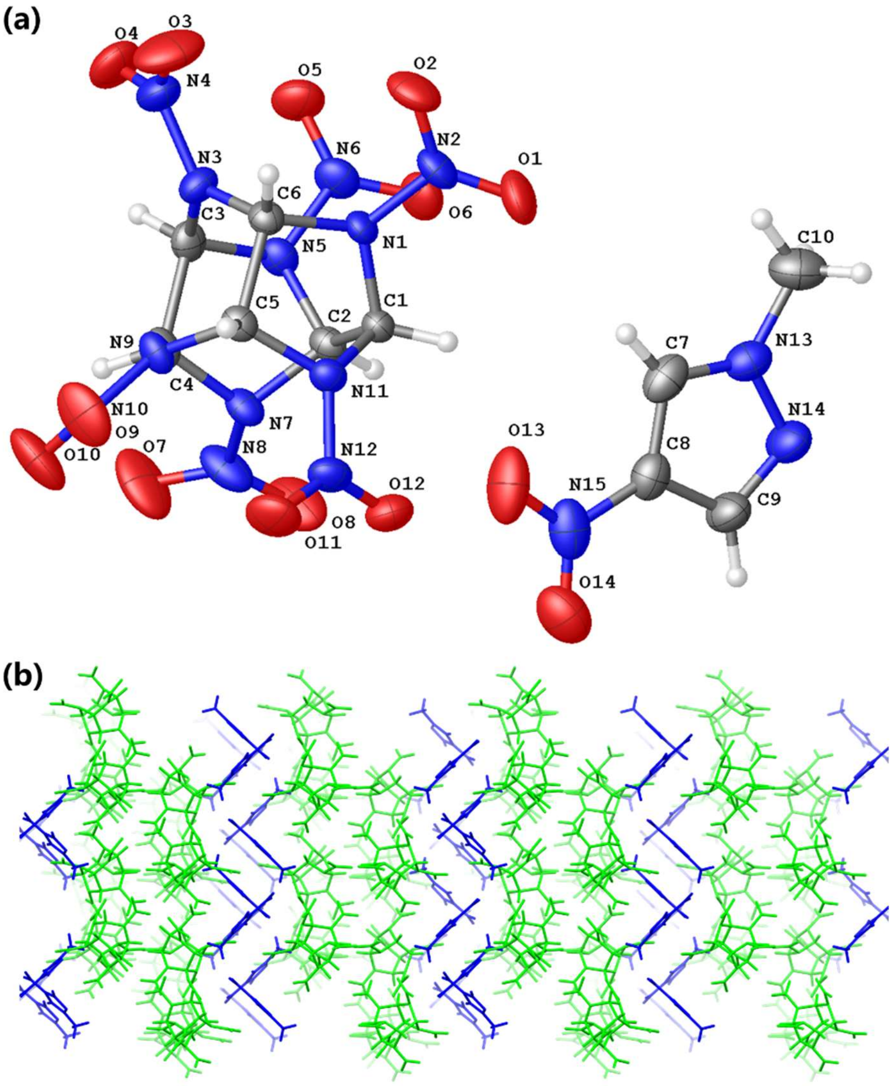

Supplementary Figure 8: Crystal structure of CL-20/1-Methyl-4-nitropyrazole.

(a) ORTEP plot of the cocrystal. **(b)** The molecular arrangement for CL-20 and 1-Methyl-4-nitropyrazole in cocrystal. Green: CL-20. Blue: 1-Methyl-4-nitropyrazole.

Supplementary Tables

Supplementary Table 1. Solvents involved in collecting cocrystal positive samples from CSD.

Toluene	4-Chlorotoluene	diglyme
DMSO-d6	1,3,5-trichlorobenzene	iodobenzene
trichloromethane-d	gamma-Butyrolactone	1,1,2-trichloroethane
ethoxyethane	DL-sec-Butyl acetate	formic acid
methylamine	iodomethane	dimethyl sulfoxide
p-Xylene	methanamide	3-methyl-1-butanol
1-butanol	Tetrahydrofuran	bromobenzene
cyclohexanone	chlorobenzene	dimethoxymethane
1H-pyrrole	Ethyl formate	2-butanone
2-butanol	isobutanol	N-Ethylmorpholine
1,1,2,2-tetrachloroethane	N, N, N', N'-Tetramethylethylenediamine	propan-2-ol
1,4-dioxane	Ethanol	2-methyl-2-propanol
2-methylpyridine	3-methylpyridine	2-butoxyethanol
diethylenetriamine	2-methoxyethanol	dibromomethane
1-methyl-2-pyrrolidone	N, N-dimethylacetamide	2,2'-Dichlorodiethyl ether
Methyl acetate	cyclopentane	benzyl alcohol
benzene	hexadecane	water-d2
nitromethane	hexamethyldisiloxane	Hexane
1-Chloro-2-Methylpropane	acetic anhydride	propanenitrile
acetamide	acetic acid	Ethylene glycol
Diethylene glycol	Isopropyl acetate	Isopropyl ether
tetrachloromethane	acetone	acetophenone
nitrobenzene	propionic acid	1,2-Propanediol
pentane	1,1-Dichloroethane	butane-1,4-diol
1,3-dimethylbenzene	1,2-dihydrostilbene	N, N-diethylethanamine
tribromomethane	2-propoxyethanol	1,2-Dichloroethane
1-propanol	water	phenylamine
heptane	trichloromethane	pyridine
cyclohexene	cyclohexane	Methanol
1,2-dimethoxyethane	3-pentanone	fluorobenzene
epichlorohydrin	acetonitrile	dichloromethane
methanedithione	1-Octanol	butanedioic acid
N, N-dimethylformamide	1,2-ethanediamine	2,4-pentanedione
o-Xylene	Propylene glycol monomethyl ether acetate	1,3,5-trimethylbenzene
2-phenylacetonitrile	2-Chlorotoluene	1,2-dichlorobenzene
isophorone	morpholine	nitric acid
quinoline	benzonitrile	ethyl acetate
benzene-d6		

Supplementary Table 2. Search spaces of hyper-parameters for CCGNet, Graph-CNN and enn-s2s.

	CCGNet		Graph-CNN		enn-s2s	
**Training**	Batch size	128	Batch size	128	Batch size	32
	Epoch	100	Epoch	100	Epoch	100
	Optimizer	Adam	Optimizer	Adam	Optimizer	Adam
**Message Passing (MP) Phase**	Number of CCGBlock	1,2,3,4,5, 6,	Number of Graph-CNN layer	1,2,3	Linear layer size	32,64,128
			Size of Graph-CNN layer	16,32,64, 128,256	Number of dense layers in $\boldsymbol{h_\Theta}$	1,2,3
	CCGBlock Size	16,32,64, 128,256	Number of Graph embedding pooling	1,2,3	Size of dense layer in $\boldsymbol{h_\Theta}$ (not out layer)	128,512, 1024,2048
					Size of output layer in $\boldsymbol{h_\Theta}$	(Linear layer size) ^2
			Size of Graph embedding pooling	8,16,32	MP step	2,3,4,5
	Activation function	ReLU, ELU, Tanh	Activation function	ReLU	Activation function	ReLU
**Readout Phase**	Readout function	Multi-head global attention	Readout function	Flatten	Readout function	Set2Set
	Number of head	Integer, [1, 20]			Processing step	2,3,4,5
	Number of dense layers	1,2,3	Number of dense layers	1,2,3	Number of dense layers	1,2,3
	Dense layer size	64,128, 256,512, 1024	Dense layer size	64,128, 256,512	Dense layer size	64,128,256, 512, 1024
	Dropout	float, [0, 0.75]	Dropout	float, [0, 0.75]	Dropout	float, [0, 0.75]
	Activation function	ReLU, ELU, Tanh	Activation function	ReLU	Activation function	ReLU
**Output**	Dense layer size	2	Dense layer size	2	Dense layer size	2

Supplementary Table 3. Search spaces of hyper-parameters for RF, SVM and DNN-desc.

<table>
<tr><th colspan="2">RF</th><th colspan="2">SVM</th><th colspan="2">DNN-desc</th></tr>
<tr><td rowspan="3">Number of estimators</td><td rowspan="3">int,
[10, 500],
step=10</td><td rowspan="3">C</td><td rowspan="3">float,
[0, 20]</td><td>Batch size</td><td>64,128,256</td></tr>
<tr><td>Epoch</td><td>100</td></tr>
<tr><td>Optimizer</td><td>Adam</td></tr>
<tr><td>Max depth</td><td>int,
[1, 20]</td><td>degree</td><td>int,
[1, 20]</td><td>Number of dense layers</td><td>1,2,3,4,5,6,7</td></tr>
<tr><td>Min samples split</td><td>int,
[2, 200]</td><td rowspan="2">gamma</td><td rowspan="2">float or string,
{[0, 1], scale, auto}</td><td>Size of dense layer</td><td>16,32,64,128,256,512</td></tr>
<tr><td>Min samples leaf</td><td>int,
[1, 50]</td><td>dropout</td><td>float, [0, 0.75]</td></tr>
<tr><td>Max features</td><td>int,
[1, 24]</td><td>coef0</td><td>float, [0, 10]</td><td>Activation function</td><td>ReLU, ELU, Tanh</td></tr>
<tr><td>Criterion</td><td>string,
{gini, entropy}</td><td rowspan="2">kernel</td><td rowspan="2">String,
{rbf, poly, sigmoid, linear}</td><td rowspan="2">Output size</td><td rowspan="2">2</td></tr>
<tr><td>Bootstrap</td><td>bool
{True, False}</td></tr>
</table>

Supplementary Table 4. The performances of all the models on the 10-fold cross-validation with and without the data augmentation. 'c1-c2' and 'c2-c1' denote two different permutations in the model input for a pair of coformers.

Model	Metrics	Data Augmentation[a]		No Data Augmentation[b]	
		c1-c2	c2-c1	c1-c2	c2-c1
SVM	TPR	99.11(±0.41)	99.11(±0.41)	99.17(±0.27)	98.51(±0.40)
	TNR	89.81(±3.55)	89.81(±3.55)	88.54(±2.52)	61.03(±4.58)
	BACC	94.46(±1.85)	94.46(±1.85)	93.85(±1.34)	79.77(±2.27)
RF	TPR	99.82(±0.15)	99.85(±0.17)	99.88(±0.11)	99.73(±0.20)
	TNR	87.05(±3.87)	86.43(±3.59)	86.05(±3.48)	72.36(±3.77)
	BACC	93.44(±1.89)	93.14(±1.75)	92.97(±1.71)	86.04(±1.87)
DNN-desc	TPR	99.55(±0.19)	99.21(±0.26)	99.59(±0.41)	98.36(±0.99)
	TNR	89.11(±2.42)	89.80(±2.95)	89.66(±4.13)	65.16(±5.49)
	BACC	94.33(±1.25)	94.51(±1.51)	94.62(±1.91)	81.76(±2.48)
DNN-FP	TPR	98.57(±0.46)	98.51(±0.32)	99.17(±0.35)	97.38(±0.37)
	TNR	86.48(±4.86)	89.07(±3.73)	90.73(±3.95)	44.71(±6.73)
	BACC	92.52(±2.37)	93.79(±1.92)	94.95(±1.98)	71.05(±3.40)
enn-s2s	TPR	98.63(±0.38)	98.63(±0.38)	98.74(±0.47)	98.74(±0.47)
	TNR	89.90(±4.98)	89.90(±4.98)	88.14(±3.67)	88.14(±3.67)
	BACC	94.27(±2.41)	94.27(±2.41)	93.44(±1.83)	93.44(±1.83)
Graph-CNN	TPR	98.94(±0.39)	98.94(±0.39)	98.91(±0.41)	98.91(±0.41)
	TNR	87.20(±3.33)	87.20(±3.33)	87.05(±4.18)	87.05(±4.18)
	BACC	93.07(±1.60)	93.07(±1.60)	92.98(±2.07)	92.98(±2.07)
GCN	TPR	98.98(±0.43)	98.47(±0.32)	99.08(±0.40)	97.37(±0.66)
	TNR	87.64(±3.47)	85.38(±5.10)	89.78(±3.77)	43.25(±6.68)
	BACC	93.31(±1.76)	91.93(±2.62)	94.43(±1.86)	70.31(±3.35)
CCGNet	TPR	99.82(±0.14)	99.78(±0.15)	99.78(±0.21)	99.24(±0.49)
	TNR	97.26(±1.61)	96.85(±1.45)	96.79(±1.35)	90.89(±3.55)
	BACC	98.54(±0.79)	98.31(±0.70)	98.28(±0.65)	95.07(±1.70)

[a] The model is trained on the inputs of c1-c2 and c2-c1.

[b] The model is trained only on the input of c1-c2.

Supplementary Table 5. Positive and negative co-crystal samples for nicotinamide in the independent test set.

Name	SMILES	Cocrystal	Ref
Fumaric Acid	O=C(O)/C=C/C(=O)O	Yes	EDAPOQ
Isonicotinamide	NC(=O)c1ccncc1	Yes	UMUYOR
Quercetin	O=c1c(O)c(-c2ccc(O)c(O)c2)oc2cc(O)cc(O)c12	Yes	NAFYUR
Adipic Acid	O=C(O)CCCCC(=O)O	Yes	NUKYIC
Isoliquiritigenin	O=C(/C=C/c1ccc(O)cc1)c1ccc(O)cc1O	Yes	EYUGAI
3-Hydroxybenzoic Acid	O=C(O)c1cccc(O)c1	Yes	XAQQIQ
Anthranilic Acid	Nc1ccccc1C(=O)O	Yes	FEBSIR
Hydrochlorothiazide	NS(=O)(=O)c1cc2c(cc1Cl)NCNS2(=O)=O	Yes	PIRXUL
P-Coumaric Acid	O=C(O)/C=C/c1ccc(O)cc1	Yes	SOLBEC
Caffeic Acid Phenethyl Ester	O=C(/C=C/c1ccc(O)c(O)c1)OCCc1ccccc1	Yes	ETONIM
Tegafur	O=c1[nH]c(=O)n(C2CCCO2)cc1F	Yes	DOXDUR
Niflumic Acid	O=C(O)c1cccnc1Nc1cccc(C(F)(F)F)c1	Yes	EXAQEA
Ethylparaben	CCOC(=O)c1ccc(O)cc1	Yes	GOGQID
Febuxostat	Cc1nc(-c2ccc(OCC(C)C)c(C#N)c2)sc1C(=O)O	Yes	HIQQIJ
Mefenamic Acid	Cc1cccc(Nc2ccccc2C(=O)O)c1C	Yes	EXAQOK
DL-Ascorbic Acid	O=C1OC(C(O)CO)C(O)=C1O	Yes	OXOHEQ
Carbamazepine	NC(=O)N1c2ccccc2C=Cc2ccccc21	Yes	UNEZES
Tranilast	COc1ccc(/C=C/C(=O)Nc2ccccc2C(=O)O)cc1OC	Yes	KINTUY
(+/-)-Ivabradine	COc1cc2c(cc1OC)CC(=O)N(CCCN(C)CC1Cc3c c(OC)c(OC)cc31)CC2	No	[1]
Mls000678252	CC(=O)SC1CC2=CC(=O)CCC2(C)C2CCC3(C) C(CCC34CCC(=O)O4)C12	No	[2]
Nitazoxanide	CC(=O)Oc1ccccc1C(=O)Nc1ncc([N+](=O)[O-])s1	No	[3]
Ehrlich's reagent	CN(C)c1ccc(C=O)cc1	No	[4]
Famotidine	NC(N)=Nc1nc(CSCC/C(N)=N/S(N)(=O)=O)cs1	No	[5]
4-Methylbenzamide	Cc1ccc(C(N)=O)cc1	No	[6]
3-Methoxybenzamide	COc1cccc(C(N)=O)c1	No	[6]
2-Methoxybenzamide	COc1ccccc1C(N)=O	No	[6]
2-Methylbenzamide	Cc1ccccc1C(N)=O	No	[6]
Acetanilide	CC(=O)Nc1ccccc1	No	[4]
4-Methoxybenzoic Acid	COc1ccc(C(=O)O)cc1	No	[6]
3-Fluorobenzoic Acid	O=C(O)c1cccc(F)c1	No	[6]
3-Nitrobenzoic Acid	O=C(O)c1cccc([N+](=O)[O-])c1	No	[6]
Indole	c1ccc2[nH]ccc2c1	No	[7]

Supplementary Table 6. Positive and negative co-crystal samples for carbamazepine in the independent test set.

Name	SMILES	Cocrystal	Ref
4-Aminobenzoic Acid	Nc1ccc(C(=O)O)cc1	Yes	XAQRAJ
4-Hydroxybenzamide	NC(=O)c1ccc(O)cc1	Yes	SOGSEP
Hydroquinone	Oc1ccc(O)cc1	Yes	ABOQUF
Salicylic Acid	O=C(O)c1ccccc1O	Yes	MOXWAY
P-Benzoquinone	O=C1C=CC(=O)C=C1	Yes	UNEYOB
Nicotinamide	NC(=O)c1cccnc1	Yes	UNEZES
4-Nitropyridine N-Oxide	O=[N+]([O-])c1cc[n+]([O-])cc1	Yes	JIQKUS
Schembl5894079	ON1C=CN(O)c2ccccc21	Yes	VIGGOI
Fumaric Acid	O=C(O)/C=C/C(=O)O	Yes	WEYFEN
2,2,2-Trifluoroethanol	OCC(F)(F)F	Yes	SAPDUJ
Thiosaccharin	O=S1(=O)NC(=S)c2ccccc12	Yes	YAJGEY
Benzoic Acid	C1=CC=C(C=C1)C(=O)O	Yes	MOXVAX
Anthranilic Acid	C1=CC=C(C(=C1)C(=O)O)N	Yes	RUTGOE
Ketoprofen	CC(C(=O)O)c1cccc(C(=O)c2ccccc2)c1	Yes	RAFGEO
2-Hydroxyacetamide	NC(=O)CO	No	[2]
Lactamide	CC(O)C(N)=O	No	[2]
Glycolic Acid	O=C(O)CO	No	[8]
DL-Tartaric Acid	O=C(O)C(O)C(O)C(=O)O	No	[8]
Methylglucamine	CNCC(O)C(O)C(O)C(O)CO	No	[2]
Malic Acid	O=C(O)CC(O)C(=O)O	No	[8]
Malic Acid	O=C(O)CC(O)C(=O)O	No	[8]
Picolinamide	NC(=O)c1ccccn1	No	[9]

Supplementary Table 7. Positive and negative co-crystal samples for indomethacin in the independent test set.

Coformer	SMILES	Cocrystal	Ref
Nicotinamide	NC(=O)c1cccnc1	Yes	SESKUY
Saccharin	O=C1NS(=O)(=O)c2ccccc21	Yes	UFERED
Neotam	COC(=O)C(Cc1ccccc1)NC(=O)C(CC(=O)O)NCCC(C)(C)C	No	[10]
Vanillic Acid	COc1cc(C(=O)O)ccc1O	No	[10]
Cyclamic Acid	O=S(=O)(O)NC1CCCCC1	No	[10]
Glycine	NCC(=O)O	No	[10]
Oxalic Acid	O=C(O)C(=O)O	No	[10]
Fumaric Acid	C(=CC(=O)O)C(=O)O	No	[10]
Citric Acid	O=C(O)CC(O)(CC(=O)O)C(=O)O	No	[10]
Malic Acid	O=C(O)CC(O)C(=O)O	No	[10]
4-Aminobenzoic Acid	Nc1ccc(C(=O)O)cc1	No	[10]
Maleic Acid	O=C(O)/C=C\C(=O)O	No	[10]
Malonic Acid	O=C(O)CC(=O)O	No	[10]
Glutaric Acid	O=C(O)CCCC(=O)O	No	[10]
Urea	NC(N)=O	No	[10]
4-Aminobenzamide	NC(=O)c1ccc(N)cc1	No	[10]
4-Hydroxybenzoic Acid	O=C(O)c1ccc(O)cc1	No	[10]
Benzoic Acid	O=C(O)c1ccccc1	No	[10]
Succinic Acid	O=C(O)CCC(=O)O	No	[10]

Supplementary Table 8. Positive and negative co-crystal samples for paracetamol in the independent test set.

Coformer	SMILES	Cocrystal	Ref
Phenazine	c1ccc2nc3ccccc3nc2c1	Yes	LUJSOZ
Theophylline	Cn1c(=O)c2[nH]cnc2n(C)c1=O	Yes	KIGLUI01
Oxalic Acid	O=C(O)C(=O)O	Yes	LUJTAM
Naphthalene	c1ccc2ccccc2c1	Yes	LUJSIT
Saccharin	O=C1NS(=O)(=O)c2ccccc21	No	[11]
DL-Ascorbic Acid	O=C1OC(C(O)CO)C(O)=C1O	No	[12]
Theobromine	Cn1cnc2c1c(=O)[nH]c(=O)n2C	No	[11]
Malonic Acid	O=C(O)CC(=O)O	No	[11]
Anthracene	c1ccc2cc3ccccc3cc2c1	No	[13]
Nicotinamide	NC(=O)c1cccnc1	No	[14]
Succinic Acid	O=C(O)CCC(=O)O	No	[11]
Adipic Acid	O=C(O)CCCCC(=O)O	No	[11]

Supplementary Table 9. Cocrystal samples (positive and negative ones) in the independent test set for Pyrene.

Coformer	SMILES	Cocrystal	Ref
1,3,5-Trifluoro-2,4,6-Triiodobenzene	Fc1c(I)c(F)c(I)c(F)c1I	Yes	QEVWEW
Pyrazine-2,3,5,6-Tetracarbonitrile	N#Cc1nc(C#N)c(C#N)nc1C#N	Yes	BORPII
1,4-Dibromotetrafluorobenzene	Fc1c(F)c(Br)c(F)c(F)c1Br	Yes	GUQRAN
N-[2-(3-Methoxypropyl)Phenyl]Cyclohexanecarboxamide	N#Cc1c(O)c(C#N)c(C#N)c(O)c1C#N	Yes	TEXPOB
Naphthalene-1,4,5,8-Tetrone	O=c1ccc(=O)c2c(=O)ccc(=O)c1=2	Yes	CEKBUP
1,4-Diiodotetrafluorobenzene	Fc1c(F)c(I)c(F)c(F)c1I	Yes	FARNOD
Chlorothalonil	N#Cc1c(Cl)c(Cl)c(Cl)c(C#N)c1Cl	Yes	HAYYOW
Iodopentafluorobenzene	Fc1c(F)c(F)c(I)c(F)c1F	Yes	GUQQIU
Tetrachloroterephthalonitrile	N#Cc1c(Cl)c(Cl)c(C#N)c(Cl)c1Cl	Yes	WAWPAM
1,3-Dibromotetrafluorobenzene	Fc1c(F)c(Br)c(F)c(Br)c1F	Yes	GUQQUG
F4-Tcnq	N#CC(C#N)=c1c(F)c(F)c(=C(C#N)C#N)c(F)c1F	Yes	MIDDEL
1,2-Dibromotetrafluorobenzene	Fc1c(F)c(F)c(Br)c(Br)c1F	Yes	GUQQOA
Hexafluorobenzene	Fc1c(F)c(F)c(F)c(F)c1F	Yes	ZZZGKE01
1,3,5-Tribromo-2,4,6-Trifluorobenzene	Fc1c(Br)c(F)c(Br)c(F)c1Br	Yes	QEVXOH
P-Benzoquinone	O=C1C=CC(=O)C=C1	Yes	PYRBZQ
4,5-Dibromophthalic Anhydride	O=C1OC(=O)c2cc(Br)c(Br)cc21	Yes	EHESUF
Picric Acid	O=[N+]([O-])c1cc([N+](=O)[O-])c(O)c([N+](=O)[O-])c1	Yes	PYRPCT02
Bromopentafluorobenzene	Fc1c(F)c(F)c(Br)c(F)c1F	Yes	GUQQEQ
2309-49-1	Cn1c(=O)c2c(n(C)c1=O)n(C)c(=O)n2C	Yes	MURPYR
Tetrafluoro-1,4-Benzoquinone	O=C1C(F)=C(F)C(=O)C(F)=C1F	Yes	PYRFLR
Tetracyanoethylene	N#CC(C#N)=C(C#N)C#N	Yes	PYRCYE10
4,7-Dibromobenzo(C)Furazan 1-Oxide	Brc1c2=NON(=c2c(Br)cc1)O	Yes	AYEGAM
917-23-7	C1=Cc2nc1c(-c1ccccc1)c1ccc([nH]1)c(-c1ccccc1)c1nc(c(-c3ccccc3)c3ccc([nH]3)c2-c2ccccc2)C=C1	Yes	XAGMAT
Hexachlorobenzene	Clc1c(Cl)c(Cl)c(Cl)c(Cl)c1Cl	Yes	ISISAG
Benzene-1,2,4,5-Tetracarbonitrile	N#Cc1cc(C#N)c(C#N)cc1C#N	Yes	PYRCBZ
Chloranil	O=C1C(Cl)=C(Cl)C(=O)C(Cl)=C1Cl	Yes	PYRCLN
Tetrachlorophthalonitrile	N#Cc1c(Cl)c(Cl)c(Cl)c(Cl)c1C#N	Yes	PINJUU02

Octafluoronaphthalene	Fc1c(F)c(F)c2c(F)c(F)c(F)c(F)c2c1F	Yes	ECUVIH
9-(4-Methoxyphenyl)Xanthen-9-Ol	COc1ccc(C2(O)c3ccccc3Oc3ccccc32)cc1	Yes	XETTEW
1487-82-7	Cc1cc(=C(C#N)C#N)c(C)cc1=C(C#N)C#N	Yes	VOQCUC
34151-49-0	O=C1c2ccc3c4c(ccc(c24)C(=O)N1c1ccncc1)C(=O)N(c1ccncc1)C3=O	Yes	GUMNUY
N,-N'-Dimethylpyromellitic Acid Diimide	Cn1c(=O)c2cc3c(=O)n(C)c(=O)c3cc2c1=O	Yes	PAYYOG
N,N'-(1,3,6,8-Tetraoxo-1,3,6,8-Tetrahydrobenzo[Lmn][3,8]Phenanthroline-2,7-Diyl)Diisonicotinamide	O=C(NN1C(=O)c2ccc3c4c(ccc(c24)C1=O)C(=O)N(NC(=O)c1ccncc1)C3=O)c1ccncc1	Yes	OPUQUN
Nsc97081	CCN1C(=O)/C(=C2\SC(=S)N(CC)C2=O)SC1=S	Yes	QOLQOA
2-Chloro-1,4-Benzoquinone	O=C1C=CC(=O)C(Cl)=C1	Yes	CORPIJ
3-Propyl-5-[3-Propyl-2,4-Bis(Sulfanylidene)-1,3-Thiazolidin-5-Ylidene]-1,3-Thiazolidine-2,4-Dithione	CCCN1C(=S)S/C(=C2/SC(=S)N(CCC)C2=S)C1=S	Yes	QOLQEQ
2-Chloro-11,11,12,12-Tetracyanoanthraquinodimethane	N#CC(C#N)=c1c2ccccc2c(=C(C#N)C#N)c2cc(Cl)ccc12	Yes	GAFJAY
Bromanil	O=C1C(Br)=C(Br)C(=O)C(Br)=C1Br	Yes	REDFIP
1518-16-7	N#CC(C#N)=c1ccc(=C(C#N)C#N)cc1	Yes	BITBUD
O-Chloranil	O=C1C(=O)C(Cl)=C(Cl)C(Cl)=C1Cl	Yes	REQVOZ
2-(3,4-Diethoxy-2,5-Dioxo-3-Cyclopenten-1-Ylidene)-Malononitrile	CCOC1=C(OCC)C(=O)C(=C(C#N)C#N)C1=O	Yes	BEFGIC
5,6-Dibromobenzofurazane 1-Oxide	[O-][n+]1onc2cc(Br)c(Br)cc21	Yes	EHESIT
Zinc361240	Cc1ccc(S(=O)(=O)N=C2C=C(Cl)C(=O)C(Cl)=C2)cc1	Yes	PYTQIM
2-Bromo-1,3,5-Trinitrobenzene	O=[N+]([O-])c1cc([N+](=O)[O-])c(Br)c([N+](=O)[O-])c1	Yes	PYRBPC
Chloropentafluorobenzene	Fc1c(F)c(F)c(Cl)c(F)c1F	Yes	GUQQAM
98507-06-3	CC1=C/C(=N\C#N)C(C)=C/C1=N\C#N	Yes	VOQDAJ
9,9'-Bifluorenylidene	c1ccc2c(c1)C(=C1c3ccccc3-c3ccccc31)c1ccccc1-2	Yes	CUNWUD
Pyromellitic Dianhydride	O=c1oc(=O)c2cc3c(=O)oc(=O)c3cc12	Yes	PYRPMA01
3-Ethyl-5-(3-Ethyl-2-Oxo-4-Sulfanylidene-1,3-Thiazolidin-5-Ylidene)-4-Sulfanylidene-1,3-Thiazolidin-2-One	CCN1C(=O)S/C(=C2/SC(=O)N(CC)C2=S)C1=S	Yes	QOLRER

3-Ethyl-5-[3-Ethyl-2,4-Bis(Sulfanylidene)-1,3-Thiazolidin-5-Ylidene]-1,3-Thiazolidine-2,4-Dithione	CCN1C(=S)S/C(=C2/SC(=S)N(CC)C2=S)C1=S	Yes	QOLPUF
3,3',5,5'-Tetrabromo-1,1'-Bi(Cyclohexa-2,5-Dien-1-Ylidene)-4,4'-Dione	O=C1C(Br)=CC(=C2C=C(Br)C(=O)C(Br)=C2)C=C1Br	Yes	BAZCUA
13-Methyl-6,7,8,9,11,12,14,15,16,17-Decahydrocyclopenta[A]Phenanthrene-3,17-Diol	CC12CCC3c4ccc(O)cc4CCC3C1CCC2O	Yes	CUTBEZ
3,3'5,5'-Tetrachlorodiphenoquinone	O=C1C(Cl)=CC(=C2C=C(Cl)C(=O)C(Cl)=C2)C=C1Cl	Yes	BAZDAH
1,8-Dinitroanthraquinone	O=C1c2cccc([N+](=O)[O-])c2C(=O)c2c1cccc2[N+](=O)[O-]	Yes	AGORAS
Methyl Picrate	COc1c([N+](=O)[O-])cc([N+](=O)[O-])cc1[N+](=O)[O-]	Yes	CILRAQ
17-Acetyl-10,13-Dimethyl-1,2,6,7,8,9,11,12,14,15,16,17-Dodecahydrocyclopenta[A]Phenanthren-3-One	CC(=O)C1CCC2C3CCC4=CC(=O)CCC4(C)C3CCC12C	Yes	CUSZUM
9,10-Dicyanoanthracene	N#Cc1c2ccccc2c(C#N)c2ccccc12	Yes	EHUFEV
6H-Benzo[C]Chromen-6-One	O=c1oc2ccccc2c2ccccc12	Yes	EHUFIZ
Carbazole	c1ccc2c(c1)[nH]c1ccccc12	No	[15]
Anthracene	c1ccc2cc3ccccc3cc2c1	No	[16]
Chrysene	c1ccc2c(c1)ccc1c3ccccc3ccc21	No	[15]
Phenanthrene	c1ccc2c(c1)ccc1ccccc12	No	[15]
Naphthalene	c1ccc2ccccc2c1	No	[15]
Fluoranthene	c1ccc2c(c1)-c1cccc3cccc-2c13	No	[15]

Supplementary Table 10. Performances of all the models on the independent test sets of TNT and CL-20 without the transfer learning.

	Metrics	SVM	RF	DNN-desc	DNN-FP	enn-s2s	Graph-CNN	GCN	CCGNet
**TNT**	**TPR (%)**	78.26	69.57	95.65	0	65.22	65.22	60.87	95.65
	**TNR (%)**	12.50	12.50	25	65.22	37.5	37.5	37.5	25
	**BACC (%)**	45.38	41.03	60.33	32.61	51.36	51.36	49.18	60.33
**CL-20**	**TPR (%)**	77.78	83.33	100	0	100	100	100	100
	**TNR (%)**	16.67	33.33	16.67	100	0	0	0	0
	**BACC (%)**	47.22	58.33	58.33	50	50	50	50	50

Supplementary Table 11. Refcodes for 116 positive samples of energetic cocrystals used in this work, collected from Cambridge Structural Database.

REDCIM	LUTGUD	POCVIP	XIZCER	ABUNIU	PYRTNB
JABYIX	SUGCAY	WIFYAN	PUTXAF	REDDAF	HETTOS
RULLUF	ZEVNUL	RENPUV	PUTXEJ	ZUBNUH	ZEGKIF10
ANCTNB	ZASWAT	SERZIB	WOSFOB	QOSRUN	YOJXON
CAZTBZ01	DUKBOC	APANBZ	KUMYOI	PUTWOS	ABTNBA01
KOBFIQ	VIGLEC	WOJXEY	VAZBIJ	BOXTET	YOJQOG
JOCTAZ	ZEZHAP	NIKLOL	GEXMIH	JAQVOP	POSREV
VIGKIF	WOJWIB	FUFSOQ	SKTNIB	ROSMOD	QARQUY
UGUNAN	ZILMUF	UTEJAG	WIFXUG	GEXMON	NILCET
GOWHIL	BNZTNB	WUGWAY	RUYLAY	FONJAV	AJAKOL
PUTWEI	ZEZGIW	PUBWEO	CECPEF	HUZSEA	PVVBFD01
NIBZAM	XEMCID	WEPTAP	HETTUY	SOQPAQ	MEPWIQ
ZEZGOC	USEZID	STINBZ	RUYKUR	PUTWIM	FOYSUJ
PUTWUY	YOJXIH	QOWBEJ	DUKBUI	MAAZNB	ZEZHOD
KIZVAQ	MANLEV	ROSMIX	GEXMED	ERAFAE	XAHZAH
CEZFOF	HETTIM	ZOPGOC	JOCSEC	JAQXUX	URILIR
HECREM	LOKJIH	JAQXOR	ZUBNOB	TIVJUF	QAPNAZ
URILUD	VIGKUR	REDDEJ	BZATNB20	PUBMUU20	DUKCAP
ZZZAGS10	ZEZHET	REDCUY	RUYLEC	PVVBKP01	CBZTNB
WOJWOH	IDENEM				

Supplementary Table 12. The ISPE (kJ/mol) values of 864 coformer pairs computed in this work.

	RDX	HNS	TATB	DAAF	DAOAF	DATB	LLM-105	FOX-7	NQ
**1-4-DNI**	-2.81	-0.39	-0.56	-1.27	-1.41	-1.33	-0.14	-13.12	-5.37
**1-BN**	-3.11	-5.46	-3.67	-1.18	-1.48	-5.01	-2.83	-3.63	-1.80
**2-4-DNI**	-1.34	-0.25	-0.19	-2.58	-2.86	-1.25	-1.68	-15.79	-6.69
**2-4-MDNI**	-3.48	-1.14	-0.07	-1.18	-1.09	-1.09	-0.48	-11.86	-4.38
**3-4-DNP**	-1.25	-1.31	-0.09	-2.52	-2.39	-0.14	-2.75	-12.64	-6.07
**3-4-MDNP**	-6.21	-5.47	-0.83	-1.79	-1.24	-2.80	-2.33	-7.14	-2.61
**3-5-DNP**	-1.61	-0.70	-0.22	-3.15	-3.11	-0.48	-3.33	-15.22	-6.88
**3-AT**	-1.88	-2.85	-1.86	-1.58	-1.59	-2.20	-2.92	-5.50	-3.82
**4-5-MDNI**	-5.82	-5.08	-1.02	-1.29	-0.96	-2.22	-2.20	-6.24	-2.21
**4-5-MDNP**	-2.89	-2.21	-0.09	-0.74	-0.34	-0.50	-0.74	-9.46	-3.50
**4-AT**	-0.26	-0.05	-0.47	0.01	-0.36	-0.48	-0.07	-5.29	-3.86
**9-BN**	-4.89	-8.49	-7.50	-1.50	-3.36	-9.09	-4.34	-3.64	-1.90
**A12**	-6.19	-7.39	-3.17	-4.12	-3.63	-5.55	-4.58	-4.23	-2.94
**A3**	-7.39	-6.78	-2.10	-4.85	-4.04	-4.62	-4.58	-5.94	-2.52
**A4**	-8.89	-8.10	-2.84	-5.99	-5.29	-5.82	-5.93	-5.69	-3.24
**A5**	-7.70	-6.19	-3.11	-3.85	-4.17	-5.20	-4.53	-3.23	-1.74
**A7**	-7.83	-6.87	-2.17	-5.24	-4.50	-4.78	-4.98	-6.02	-2.95
**A8**	-9.85	-5.94	-1.56	-4.39	-3.47	-4.13	-4.44	-0.13	-0.08
**AA_1**	-3.06	-4.78	-3.09	-0.72	-1.40	-4.03	-2.54	-7.97	-4.70
**ADNP**	-2.13	-2.61	-0.10	-2.26	-1.97	-0.73	-2.43	-12.38	-5.16
**alpha-ABA_1**	-9.09	-10.40	-9.44	-5.13	-6.63	-10.30	-8.26	-12.34	-8.16
**ANAT**	-6.35	-5.80	-3.46	-4.73	-4.30	-4.77	-5.61	-1.65	-1.14
**Ant**	-4.27	-8.93	-6.64	-2.19	-2.47	-8.64	-3.97	-2.55	-1.65
**AT**	-0.22	-0.41	-0.06	-0.02	-0.09	-0.06	-0.11	-3.98	-2.17
**ATNO2**	-2.83	-1.91	-1.22	-2.59	-2.99	-2.00	-3.17	-6.26	-5.32
**ATZ**	-0.26	-0.05	-0.47	0.01	-0.36	-0.48	-0.07	-5.29	-3.86
**AZ1**	-1.20	-2.61	-3.61	-3.02	-3.58	-2.66	-3.72	-17.44	-6.97
**AZ2**	-1.11	-2.87	-2.65	-1.40	-1.77	-2.50	-2.51	-14.40	-6.01
**Benzene-1-2-diamine**	-2.11	-2.05	-0.06	-0.09	0.00	-0.07	-0.44	-5.61	-1.86
**BL**	-5.61	-4.37	-1.70	-4.97	-4.41	-3.44	-4.28	-7.35	-5.28
**BQ**	-5.10	-5.28	-0.55	-1.93	-1.10	-2.31	-1.77	-6.49	-2.26
**BTF**	-6.10	-5.56	-6.60	-4.07	-5.50	-6.81	-5.30	-18.41	-8.05
**BTO**	-3.19	-1.85	-3.97	-3.09	-3.43	-4.31	-1.59	-23.41	-6.78
**CE**	-1.18	-0.59	-1.67	-0.20	-0.80	-1.21	-0.13	-15.37	-4.88
**CIM**	-2.47	-0.21	-0.06	-1.32	-1.36	-0.13	-0.86	-6.56	-4.36
**CL-20**	-7.21	-7.45	-15.12	-11.52	-13.00	-12.19	-11.64	-34.22	-13.81
**CPL**	-4.26	-1.70	-0.08	-4.01	-3.87	-1.64	-3.78	-7.41	-4.88

**DADP**	-9.36	-10.79	-4.91	-3.14	-1.94	-6.72	-5.12	-2.41	-0.36
**DAF**	-3.40	-1.84	-1.96	-1.37	-1.87	-2.32	-1.53	-6.36	-5.32
**DANA**	-4.04	-3.53	-0.09	-0.98	-0.37	-0.87	-1.06	-7.83	-2.90
**DAT**	-6.03	-4.91	-1.70	-2.23	-1.74	-2.63	-2.83	-3.91	-1.91
**D-explosive**	-1.57	-0.19	-1.02	-1.09	-1.07	-1.43	-0.22	-17.03	-5.14
**DMB**	-6.46	-10.70	-4.26	-1.91	-0.95	-6.72	-3.52	-3.36	-0.68
**DMF**	-6.80	-5.19	-2.42	-6.10	-5.71	-4.33	-5.60	-7.94	-5.93
**DMI**	-8.90	-7.83	-4.09	-7.62	-7.18	-6.50	-7.95	-9.00	-6.86
**DNB**	-2.92	-1.99	-0.08	-0.75	-0.16	-0.40	-0.66	-9.64	-3.24
**DNBT**	-0.71	-0.22	-0.06	-1.68	-1.72	-0.40	-1.32	-15.72	-5.03
**DNDA**	-5.93	-5.09	-0.66	-1.69	-1.08	-2.31	-1.93	-6.62	-2.29
**DNP**	-5.93	-5.09	-0.66	-1.69	-1.08	-2.31	-1.93	-6.61	-2.29
**DNPP**	-2.78	-3.70	-1.14	-3.52	-3.64	-0.58	-4.58	-16.52	-7.21
**DNT**	-3.44	-2.69	-0.11	-0.91	-0.42	-0.38	-0.82	-9.49	-3.32
**DNTF**	-2.46	-1.41	-3.35	-1.42	-2.25	-2.79	-0.98	-17.89	-5.97
**DO**	-7.44	-7.43	-3.61	-4.42	-3.49	-5.38	-5.31	-2.76	-0.43
**EDNA**	-2.43	-3.04	-0.09	-2.50	-2.01	-0.95	-2.96	-11.43	-5.16
**FA**	-7.68	-4.15	-1.07	-5.45	-4.99	-3.68	-4.25	-6.14	-5.50
**GTA**	-10.94	-11.11	-4.09	-5.33	-4.16	-7.45	-5.31	-4.21	-1.24
**HMX**	-0.38	-0.65	-0.15	-0.05	-0.12	-0.62	-0.15	-10.95	-3.22
**MAM**	-5.67	-6.53	-1.78	-2.27	-1.44	-3.53	-3.06	-3.24	-0.88
**MATNB**	-1.47	-1.90	-0.20	-0.64	-0.35	-0.55	-0.68	-11.37	-4.46
**MDNT**	-2.51	-0.37	-0.23	-0.62	-0.63	-0.74	-0.21	-13.60	-4.61
**MNO**	-2.72	-1.62	-0.12	-1.45	-1.15	-1.06	-0.87	-11.93	-4.28
**MTNP**	-1.50	-0.44	-0.81	-0.11	-0.43	-0.62	-0.45	-12.28	-4.28
**Nap**	-4.57	-9.63	-6.72	-2.37	-2.64	-8.68	-4.29	-2.01	-1.55
**NAQ**	-4.78	-4.96	-1.01	-2.48	-1.80	-2.85	-2.68	-5.39	-1.74
**NMP**	-8.72	-6.77	-4.01	-7.85	-7.34	-6.00	-7.87	-8.27	-6.67
**NN**	-5.84	-5.59	-1.48	-3.16	-2.56	-3.31	-3.21	-4.91	-1.74
**NTO**	-1.46	-0.56	-0.76	-2.53	-2.72	-0.96	-2.43	-15.44	-6.76
**PA**	-1.57	-0.19	-1.02	-1.09	-1.07	-1.43	-0.22	-17.03	-5.14
**PDA**	-2.30	-2.24	-0.25	-0.28	-0.19	-0.26	-0.63	-5.80	-2.06
**PDCA**	-6.22	-8.32	-5.97	-2.85	-3.44	-6.80	-5.20	-6.45	-3.97
**Per**	-4.99	-9.26	-6.81	-2.41	-3.06	-8.75	-4.60	-3.91	-2.36
**PETN**	-3.50	-3.12	-3.34	-1.63	-2.83	-3.95	-2.16	-14.87	-5.09
**phenanthrene**	-3.28	-8.17	-5.00	-1.34	-1.46	-6.99	-2.91	-2.96	-2.28
**Pnox**	-10.93	-8.90	-3.43	-8.55	-7.85	-6.81	-7.69	-6.70	-5.37
**Py**	-3.86	-2.66	-0.81	-2.02	-1.59	-1.75	-1.88	-1.93	-0.28
**Pyr**	-1.80	-5.54	-2.96	-0.14	-0.15	-4.52	-1.19	-3.24	-0.58
**TBTNB**	-1.32	-0.76	-1.01	-0.37	-0.81	-1.16	-0.95	-12.01	-3.44
**TCTNB**	-0.89	0.06	0.28	-0.16	-0.44	-0.45	-0.66	-10.73	-3.17
**TITNB**	-2.53	-4.19	-4.03	-1.13	-1.89	-4.14	-2.68	-12.98	-4.45
**TNA**	-0.96	-0.47	-0.42	-0.58	-0.55	-0.69	-0.52	-14.80	-4.84
**TNAN**	-1.01	-0.63	-0.18	-0.67	-0.55	-0.69	0.03	-13.04	-4.02

**TNAZ**	-1.44	-0.52	-2.94	-1.01	-1.97	-2.65	-1.22	-18.76	-6.11
**TNB**	-1.61	-1.00	-0.93	-0.91	-1.31	-1.74	-0.79	-12.98	-4.17
**TNGU**	-5.17	-5.83	-7.71	-6.10	-8.40	-7.69	-8.78	-22.30	-9.57
**TNT**	-0.91	-0.24	-0.50	-0.60	-0.55	-0.77	0.01	-14.42	-4.35
**TTNB**	-3.43	-4.83	-6.16	-0.70	-1.83	-7.54	-2.36	-9.02	-3.28
**TZTN**	-4.07	-1.94	-0.69	-3.01	-3.17	-2.04	-3.06	-11.61	-6.02
**xylene**	-3.68	-5.88	-3.47	-0.96	-0.79	-4.48	-2.93	-1.64	-0.02
**RDX**	--	-1.33	-3.07	-0.78	-0.85	-2.09	-0.91	-19.28	-5.41
**HNS**	-1.33	--	-1.05	-1.23	-1.21	-1.60	0.18	-17.16	-4.86
**TATB**	-3.07	-1.05	--	-0.55	-0.10	-0.27	-0.27	-9.17	-2.86
**DAAF**	-0.78	-1.23	-0.55	--	-0.03	-0.42	-0.37	-10.41	-4.22
**DAOAF**	-0.85	-1.21	-0.10	-0.03	--	-0.08	-0.08	-9.36	-3.72
**DATB**	-2.09	-1.60	-0.27	-0.42	-0.08	--	0.08	-13.25	-3.90
**LLM-105**	-0.91	0.18	-0.27	-0.37	-0.08	0.08	--	-11.50	-4.12
**FOX-7**	-19.28	-17.16	-9.17	-10.41	-9.36	-13.25	-11.50	--	-1.65
**NQ**	-5.41	-4.86	-2.86	-4.22	-3.72	-3.90	-4.12	-1.65	--

Supplementary Table 13. Performance of the best 10 CCGNet models.

Model	Loss	Valid Accuracy
1	0.00037	1.0
2	0.00042	1.0
3	0.00055	1.0
4	0.00122	1.0
5	0.00272	1.0
6	0.00368	1.0
7	0.00412	1.0
8	0.00673	1.0
9	0.00977	1.0
10	0.02454	1.0

Supplementary Table 14. Heats of formation for H, C, O and N atoms.

Atoms	$H^{\circ}_{(\text{atoms},298)}[\text{a.u.}]^{a}$ (Hartree/Particle)	$\Delta H^{\circ}_{f\ (\text{atoms},298)}{}^{b}$ (kJ/mol)
H	-0.500991	218.2
C	-37.786156	717.2
O	-74.991202	249.5
N	-54.522462	473.1

[a] the calculated values by CBS-4M method
[b] the experimental values[17]

Supplementary Table 15. The impact sensitivity and heat of explosion calculated for coformers 5, 7, 8, 10.

**Cofomers**	**Impact Sensitivity (IS)**	**Heat of explosion** (Q, cal/g)
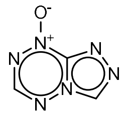 Coformer 5	1.5941	1.483×10^{3}
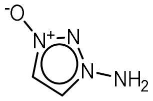 Coformer 7	2. 3146	1.484×10^{3}
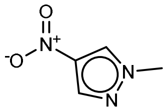 Coformer 8	2.8939	1.174×10^{3}
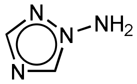 Coformer 10	6.2555	0.797×10^{3}

Supplementary Table 16. Crystal data and structure refinement for CL-20/1-Methyl-4-nitropyrazole cocrystal.

**CCDC no.**	2107286
**Empirical formula**	$C_{10}H_{11}N_{15}O_{14}$
**Formula weight**	565.34
**Temperature**	296 K
**Crystal system**	Orthorhombic
**Space group**	Pbca
**Unit cell dimensions**	a = 11.9344(12) Å a= 90°<br>b = 10.9427(10) Å b= 90°<br>c = 31.436(3) Å γ = 90°
**Volume**	4105.4(7) $Å^3$
**Z**	8
**Density (calculated)**	1.829 g/cm^3
**Absorption coefficient**	0.170 mm^{-1}
**F(000)**	2304
**Crystal size**	0.33 x 0.3 x 0.28 mm^3
**Theta range for data collection**	2.143 to 30.466°.
**Index ranges**	-16<=h<=17, -15<=k<=11, -44<=l<=44
**Reflections collected**	39289
**Independent reflections**	6237 [R(int) = 0.0290]
**Completeness to theta = 25.242°**	99.90%
**Absorption correction**	Semi-empirical from equivalents
**Max. and min. transmission**	0.7461 and 0.6897
**Refinement method**	Full-matrix least-squares on F^2
**Data / restraints / parameters**	6237/0/353
**Goodness-of-fit on F^2**	1.043
**Final R indices [I>2sigma(I)]**	R1 = 0.0480, wR2 = 0.1171
**R indices (all data)**	R1 = 0.0640, wR2 = 0.1282
**Extinction coefficient**	n/a
**Largest diff. peak and hole**	0.312 and -0.351 $e.Å^{-}$

Supplementary Discussion

Attention Visualization

The interpretability of the deep learning model has been a challenge. Herein, we introduce the attention mechanism to further optimize the feather space on one side, and make the model interpretable on the other side. The attention can weight important patterns associated with the target property. Supplementary Figure 1 representatively selects some cocrystals to exemplify, where the attention weights are mapped to corresponding atoms in 2D structures, in turn visualizing some important patterns hidden under the data. For WAWDEE (Cambridge Structural Database refcode) in Supplementary Figure 1(a), the attention weights highlight on the nitrogen atoms and hydroxy groups involving the intermolecular H-bonding and the benzene ring involving the weak intermolecular π-π interaction with the pyrimidine ring. The crystal structure of KARHAM shows that the co-crystalization is mainly driven by the intermolecular π-π stack while the attention coefficients just capture these groups involving the interaction, as reflected by the 2D structure in Supplementary Figure 1(b). The other examples in Supplementary Figure 1 similarly reflect that the attention weights can capture the atoms and groups involving the important interactions between a pair of cofomers.

Supplementary Methods

Construction and Bayesian optimization of the competitive models

In the section, we briefly describe the construction of the seven competitive models and Bayesian optimization on their hyder-parameters, along with their best configurations determined by the optmization.

RF and SVM are conventional machine learning (ML) models. RF (Random Forest) is an ensemble learning method that contains multiple decision trees. For the classification task, the output of RF is the class selected by most trees. SVM (support-vector machine) is a supervised learning model for the classification and regression. SVM maps training samples to points in space so as to maximize the width of the gap between the two classes. New samples are then mapped into that same space and predicted to belong to a class based on which side of the gap they fall. We implement RF and SVM by using Scikit-learn[18].

DNN-FP and DNN-desc are Deep Neural Networks composed of dense layers. DNN-FP is also derived from the work of Devogelaer et al[19] and used the extended-connectivity fingerprints (ECFP) as the feature descriptor. Following Ref.[19], we build its framework (see Ref.[19] for more details). For DNN-desc, it is constructed by sequential dense layers and adopts the concatenation of the 12 molecular descriptors of a pair of coformer as input. DNN-FP and DNN-desc are implemented by TensorFlow[20].

Graph-CNN is a spatial-based graph convolution network proposed by Felipe et al[21], where key components are Graph-CNN layers and Graph Embedding Pooling (GEP) layers. The two kinds of layers perform the message passing phase. The readout phase is a flattening operation. Excepting for the final output layer, batch normalization [22] and ReLU [23] are applied in each layer. Since the graph convolution of our CCGblock follows the Graph-CNN layer framework (see Methods in the text for details of Graph-CNN layer). Here we mainly introduce the GEP layer, as shown in Supplementary Figure 4.

Like pooling layers in conventional CNNs, the GEP layer is used to reduce dimensions of the input, which eliminates redundant information and also improves performance of computation. GEP transfers a graph with node number N to a given number N'. For this purpose, an embedding matrix $\mathbf{X}_{\text{emb}} \in \mathbb{R}^{N\times N'}$ is produced by a filter tensor $\mathbf{H}_{\text{emb}} \in \mathbb{R}^{N\times N\times C\times N'}$. The calculation of $\mathbf{X}_{\text{emb}}$ is similar to the multiple filters Graph-CNN (vide Methods), where the learnable filter $\mathbf{H}_{\text{emb}}$ is multiplied by the node features $\mathbf{X}_{\text{in}}$. It is defined by Supplementary Equations (1-2):

$$\mathbf{X}_{\text{emb}}^{(n')} = \sum_{c=1}^{C} \mathbf{H}_{\text{emb}}^{(c,n')} \mathbf{X}_{\text{in}}^{(c)} + b \tag{1}$$

$$\mathbf{X}_{\text{emb}} = \text{softmax}(\text{GConv}_{\text{emb}}(\mathbf{X}_{\text{in}}, N') + \mathbf{b}) \tag{2}$$

where $\mathbf{H}_{\text{emb}}^{(c,n')} \in \mathbb{R}^{N\times N}$ is a part of $\mathbf{H}_{\text{emb}}$. $\mathbf{X}_{\text{emb}}^{(n')}$ is a column of $\mathbf{X}_{\text{emb}} \in \mathbb{R}^{N\times N'}$. The pooled graph data will be calculated by the next operations (Supplementary Equations (3-4)).

$$\mathbf{X}_{\text{out}} = \mathbf{X}_{\text{emb}}^{\text{T}} \mathbf{X}_{\text{in}} \tag{3}$$

$$\mathbf{A}_{\text{out}} = \mathbf{X}_{\text{emb}}^{\text{T}} \mathbf{A}_{\text{in}} \mathbf{X}_{\text{emb}} \tag{4}$$

where $\mathbf{A}_{\text{in}} \in \mathbb{R}^{N\times N}$ is adjacency matrix, $\mathbf{A}_{\text{out}} \in \mathbb{R}^{N'\times N'}$ is pooled adjacency matrix. $\mathbf{X}_{\text{out}} \in \mathbb{R}^{N'\times C}$ is pooled node feature matrix. Finally, a pooled graph that has $\mathbf{A}_{\text{out}}$ and $\mathbf{X}_{\text{out}}$ is produced by GEP. Graph-CNN is built by using TensorFlow[20].

enn-s2s, proposed by Gilmer et al [24], has two phases (a message passing phase and a readout phase), as shown in Supplementary Figure 6(g). In Gilmer's work, enn-s2s is a regression model. Here, in order to extend its application to the classification prediction of the cocrystal formation, we modified the architecture of enn-s2s by changing the dimension of output layer. The message passing phase includes two functions, *i.e.*, message passing function and update function. The message passing function is used to propagate node features, as reflected by Supplementary Equation (6).

$$\mathbf{x}_i^t = \mathbf{W}\mathbf{x}_i^{t-1} + \sum_{j\in\mathcal{N}(i)} \mathbf{x}_j^{t-1} \cdot \text{MLP}(\mathbf{e}_{i,j}) \tag{5}$$

Where $\mathbf{x}_i^t$ is the feature of node i in t-th time step, $\mathbf{W}$ is trainable weights, $\mathcal{N}(i)$ is the adjacent nodes of node i, $\mathbf{e}_{i,j}$ is the feature of edge between node i and j. MLP is multi-layer perceptron.

The update function used to update node features is Gated Recurrent Unit (GRU)[25], as described by Supplementary Equation (7)

$$\mathbf{h}_i^t = \mathrm{GRU}\left(\mathbf{h}_i^{t-1}, \mathbf{x}_i^t\right) \tag{6}$$

Where $\mathbf{h}_i^t$ is the hidden state of node i in t-th time step.

For the readout phase, a feature vector for the whole graph is computed by enn-s2s that is based on iterative content-based attention from Vinyals et al [26] (Supplementary Equation 8-11)

$$\mathbf{q}_t = \mathrm{LSTM}(\mathbf{q}_{t-1}^*) \tag{7}$$

$$\alpha_{i,t} = \frac{\exp(\mathbf{x}_i \cdot \mathbf{q}_t)}{\sum_{j \in \boldsymbol{G}} \exp\,(\mathbf{x}_j \cdot \mathbf{q}_t)} \tag{8}$$

$$\mathbf{r}_t = \sum_{i=1}^{N} \alpha_{i,t}\, \mathbf{x}_i \tag{9}$$

$$\mathbf{q}_t^* = \mathbf{q}_t \parallel \mathbf{r}_t \tag{10}$$

where i indexes through each node feature vector $\mathbf{x}_i$, $\mathbf{q}_t$ is a query vector which allows us to read $\mathbf{r}_t$ from the memories at t-th time step, $\alpha_{i,t}$ is attention coefficient of node i at t-th time step, and LSTM is Long Short-Term Memory [27] which calculates a recurrent state. $\boldsymbol{G}$ is the graph to which node i and j belong. N is the number of nodes in graph $\boldsymbol{G}$. $\parallel$ is concatenation. t is the step index, which is the number of times that the state is computed. enn-s2s is implemented by Pytorch Geometric[28].

GCN was developed by Devogelaer et al for cocrystal screening [19] and its algorithm was mainly derived from Refs. [29] and [30]. The graph convolution operator of GCN trains a distinct weight matrix for each possible vertex degree, as shown in Supplementary Equation (11).

$$\mathbf{x}_i' = \mathbf{W}_1^{(\deg(i))}\mathbf{x}_i + \mathbf{W}_2^{(\deg(i))} \sum_{j \in \mathcal{N}(i)} \mathbf{x}_j \tag{11}$$

where $\deg(i)$ is the degree of node i. $\mathbf{W}_1^{(\deg(i))}$ and $\mathbf{W}_2^{(\deg(i))}$ are the trainable

weights for the node with degree $\deg(i)$. $\mathbf{x}_i$ is the feature vector of node i. $\mathcal{N}(i)$ is the neighborhoods of node i. $\mathbf{x}_j$ is the feature vectors of neighborhoods of node i. Following the GCN framework described in the work[19] (see Ref.[19] for more details), we build GCN by using DeepChem, and TensorFlow[20] is used for the backend.

Bayesian optimization is a sequential design strategy[31], which is usually employed to optimize hyper-parameters of ML models[19,32]. Thus, we, in the work, also use it to search the optimal hyper-parameters of all the models, based on our cocrystal dataset. Concretely, we randomly split cross-validation set into a training (90%) and validation (10%) set. The value of the loss function as the objective of Bayesian optimizer. In each iteration, the model is trained for 100 epochs and the optimizer optimizes the hyperparameters of model for the next iteration based on the current hyperparameters and minimum loss in the validation set. In this work, we set the number of iterations as 100. After the100 iterations, Bayesian optimizer will give the optimal hyperparameters. Supplementary Tables 2-3 list the searching space of hyper-parameters for CCGNet, Graph-CNN, enn-s2s, SVM, RF and DNN-desc. The searching spaces of DNN-FP and GCN are adopted from Devogelaer's work [19], thus not repeatedly listing them (see Ref.[19] for details). The best configuration of each model is shown in Supplementary Figure 5. Herein, Bayesian optimization is implemented by Python package Hyperopt [33].

Construction of energetic cocrystal (ECC) dataset

In order to finetune CCGNet model trained on the CC dataset to perform the transfer learning strategy, a training set composed of 243 energetic co-crystals is constructed (called as ECC dataset below), of which 116 positive samples are collected from CSD (Supplementary Table 11). Unfortunately, there are currently no public reports on failed experiments about the energetic cocrystals. Thus, we constructed the negative samples based on experimental experiences and a calculation criterion. In general, energetic molecules with strong rigidity and large steric hindrance are found to be difficult to interact with heterogeneous molecules. In addition, due to the competitive mechanism

of co-crystallization, molecules that have strong potential of homogeneous H-bonding tend to form single crystal will disfavor the formation of cocrytal between heterogeneous molecules[34,35]. Therefore, we select nine energetic molecules with these characteristics as coformers of potential negative samples for the energetic cocrystals, as shown in Supplementary Figure 7(a). We combine the nine molecules with the coformers from the positive samples of the energetic cocrystals, and initially yield 864 possible negative-samples of the energetic cocrystals. To further reduce the probability of the false negatives, we use the intermolecular site pairing energy (ISPE) proposed by Hunter et [36] as a screening criterion, which is based on the gas phase molecular electrostatic potential surfaces (MEPS) of each coformer to calculate the intermolecular interaction energy, as reflected by Supplementary Equation (12):

$$\Delta E = E_{cc} - nE_1 - mE_2 \quad (12)$$

where E_1 and E_2 are ISPE values of conformer 1 and conformer 2, respectively. E_{cc} is ISPE of the cocrystal with stoichiometry ratio of $n{:}m$ (1:1 in this work). The smaller the ΔE, the higher the probability of co-crystallization. All coformers are optimized at the level of B3LYP/6-31G (using Gaussian 09[37]) while MPES is calculated by Multiwfn [38]. The threshold of ΔE is set to be -11 kJ/mol that was adopted from Hunter et al. [36]. Supplementary Table 12 shows ISPEs of the 864 negative samples, 815 of which satisfy ΔE>-11 kJ/mol. Considering the fact that the negative samples are combined by the computation method and only 116 positive samples from CSD in the training set of the energetic co-crystals, we randomly selected 118 negative samples from the 815 computational combinations into the training set. In addition, we also add nine negative samples from fifteen failed experiments in the co-crystallization with CL-20 in our laboratory to the training set, as shown in Supplementary Figure 7(b), and the remaining six failed combination pairs are selected into the independent set. Consequently, the ECC dataset that are used to finetune CCGNet contains 116 positive samples and 127 negative ones.

Calculations of the impact sensitivity and heat of explosion to select the conformer of CL-20 for experimental synthesis

Impact sensitivity (IS) of energetic materials (EMs) is often evaluated by h_{50} that is a height of 50% probably in causing an explosion. Therefore, The higher h_{50}, the lower impact sensitivity. In this work, we used the empirical formula from Keshavarz [39] to evaluate h_{50} (Supplementary Equation 13).

$$\log(h_{50}) = \frac{52.13a + 31.80b + 117.6\sum \mathrm{SSP}_i}{\mathrm{MW}} \tag{13}$$

Where a and b are the number of C and H atoms, respectively. The SSP_i is the specific structural parameters that can decrease or increase impact sensitivity. It can be specifically calculated according to molecular structures as: 1) The existence of amino derivatives as substituents in central heteroarene; 2) Attaching an aromatic ring (e.g. picryl) to nitrogen and presence of one nitro group in specific position; 3) The attachment of an aromatic ring to nitrogen in position 1 of nitro-1,2,3-triazole explosives. However, if one of substituents in polynitroheteroarenes contains another more active site for initiation of decomposition, e.g. R-NO_2, the value of $\sum \mathrm{SSP}_i$ can be taken as zero. (see ref [39] for more details).

The heat of explosion (Q) refers to the total amount of energy released in the explosive reaction and is of great significance for gauging the explosion performance of the energetic compounds. Here Kamlet–Jacobs equations[40] are used to compute the heat of explosion Q (cal/g). For the energetic compound $\mathrm{C}_a\mathrm{H}_b\mathrm{O}_c\mathrm{N}_d$, Q can be expressed by Supplementary Equations 14-16:

$$Q = \frac{28.9b + 94.05a + 0.239\Delta H_{298K}}{M} \quad \left(if\ c > 2a + \frac{b}{2}\right) \tag{14}$$

$$Q = \frac{28.9b + 94.05\left(\frac{c}{2} - \frac{b}{4}\right) + 0.239\Delta H_{298K}}{M} \quad \left(if\ \frac{b}{2} < c < 2a + \frac{b}{2}\right) \tag{15}$$

$$Q = \frac{57.8c + 0.239\Delta H_{298K}}{M} \quad \left(if\ c < 2a + \frac{b}{2}\right) \tag{16}$$

where M is the molecular mass (g/mol) of $\mathrm{C}_a\mathrm{H}_b\mathrm{O}_c\mathrm{N}_d$. ΔH_{298K} is the heat of formation derived from the atomization energy method that breaks down molecules into atoms and uses known isolated atoms to calculate the heat of formation and CBS-4M

electronic enthalpies[41-44]. The CBS-4M electronic enthalpies are calculated by using the Gaussian 09 program[37]. The heat of formation (kJ/mol) can be calculated by the atomization energy method, as expressed by Supplementary Equation 17:

$$\Delta H_{298K} = H_{(\text{molecule},298)} - \sum H^{\circ}_{(\text{atoms},298)} + \sum \Delta H^{\circ}_{f\,(\text{atoms},298)} \qquad (17)$$

$H_{(\text{molecule},298)}$ denotes the calculated value of the heat of formation of molecules at 298K. $H^{\circ}_{(\text{atoms},298)}$ is the sum of calculated heat of formation over all atoms at 298K while $\Delta H^{\circ}_{f\,(\text{atoms},298)}$ is the sum of the experimental values of standard heat of formation over all atoms at 298K. Supplementary Table 14 lists the heat of formation values of the H, C, O and N atoms, derived from CBS-4M calculations and experiments[17].

Supplementary Table 15 lists the impact sensitivity and the heat of explosion for the coformers **5**,7,**8** and **10**. Due to the high sensitivity of CL-20, we tend to choose the coformer with the relatively low sensitivity. The higher IS, the lower sensitivity. For the four coformers, the sensitivities of the coformers **8** and **10** are lower than those of the coformers **5** and **7.** Despite the lowest sensitivity for the conformer **10**, its explosion heat Q calculated is also the lowest ($0.797 \times 10^3 \text{cal/g}$), which may disfavor the explosion performance of the cocrystal explosive. Compared to the coformer **10**, the coformer **8** has a higher explosive heat Q ($1.174 \times 10^3 \text{cal/g}$). Thus, trading off the impact sensitivity and the heat of explosion, we finally select the conformer **8** to make an attempt to synthesize its cocrystal with CL-20.

Energetic cocrystal synthesis and characterization

Material preparation. 2,4,6,8,10,12-hexanitrohexaazaisowurtzitane (ε-CL-20) was provided by the China Academy of Engineering Physics (CAEP). The 1-Methyl-4-nitropyrazole and other reagents used in this study were purchased online without further purification.

Synthesis. The raw materials ε-CL-20 (438 mg, 1 mmol) and 1-Methyl-4-nitropyrazole

(127 mg, 1 mmol) were put into a round-bottom flask with anhydrous methanol (100mL) at room temperature. Then a magnetic stirring was used to stir the mixed solution until all the solids dissolve completely. The solution was filtered into a glass bottle which was sealed with a perforated seal film. Then solvent was evaporated at room temperature. After several days, CL-20/1-Methyl-4-nitropyrazole cocrystal was obtained.

Single Crystal X-ray Diffraction (SXRD). Single crystal Xray diffraction data for the cocrystal were collected using a Bruker APEX-II-CCD diffractometer with graphite-monochromated Mo−Kα radiation (λ = 0.71073 Å). The crystal was kept at 296 K during data collection. The data were processed using Olex2 software. The structures were solved and refined with SHELXL. All non-hydrogen atoms were refined anisotropically, and hydrogen atoms were refined using the riding mode. A high quality cocrystal of CL-20/1-Methyl-4-nitropyrazole was characterized by SXRD. The crystallographic data for the cocrystal are illustrated in Supplementary Table 16. The crystal structure is shown in Supplementary Figure 8(a)-(b). Supplementary Figure 8(a) is ORTEP plot of the cocrystal. The CL-20 molecules and 1-Methyl-4-nitropyrazole molecules stack together in a sandwich-like style shown in Supplementary Figure 8(b).

Supplementary References

1 Veronika, S., Ond?Ej, D., Gregor, S., Eli?Ka, S. & Bohumil, K. Ivabradine Hydrochloride (S)-Mandelic Acid Co-Crystal: In Situ Preparation during Formulation. *Crystals* **7**, 13 (2017).

2 Yamamoto, K., Tsutsumi, S. & Ikeda, Y. Establishment of cocrystal cocktail grinding method for rational screening of pharmaceutical cocrystals. *International Journal of Pharmaceutics* **437**, 162-171, doi:https://doi.org/10.1016/j.ijpharm.2012.07.038 (2012).

3 Félix-Sonda, B. C., Rivera-Islas, J., Herrera-Ruiz, D., Morales-Rojas, H. & Höpfl, H. Nitazoxanide Cocrystals in Combination with Succinic, Glutaric, and 2,5-Dihydroxybenzoic Acid. *Crystal Growth & Design* **14**, 1086-1102, doi:10.1021/cg4015916 (2014).

4 Kant, V. Study of phase diagram and microstructures of some nicotinamide based binary organic eutectic. *Bulletin of Pharmaceutical and Medical Sciences (BOPAMS)* **1** (2013).

5 Wu, T.-k., Lin, S.-Y., Lin, H.-L. & Huang, Y.-T. Simultaneous DSC-FTIR microspectroscopy used to screen and detect the co-crystal formation in real time. *Bioorganic & Medicinal Chemistry Letters* **21**, 3148-3151, doi:https://doi.org/10.1016/j.bmcl.2011.03.001 (2011).

6 Wicker, J. G. *et al.* Will they co-crystallize? *CrystEngComm* **19**, 5336-5340 (2017).

7 Kant, V. Nicotinamide–Indole Binary Drug System: Thermodynamic and Interfacial Studies. *Molecular Crystals and Liquid Crystals* **608**, 211-222 (2015).

8 Childs, S. L. *et al.* Screening strategies based on solubility and solution composition generate pharmaceutically acceptable cocrystals of carbamazepine. *CrystEngComm* **10**, 856-864, doi:10.1039/B715396A (2008).

9 Habgood, M., Deij, M. A., Mazurek, J., Price, S. L. & ter Horst, J. H. Carbamazepine Co-crystallization with Pyridine Carboxamides: Rationalization by Complementary Phase Diagrams and Crystal Energy Landscapes. *Crystal Growth & Design* **10**, 903-912, doi:10.1021/cg901230b (2010).

10 Mohammad, M. A., Alhalaweh, A. & Velaga, S. P. Hansen solubility parameter as a tool to predict cocrystal formation. *International Journal of Pharmaceutics* **407**, 63-71, doi:https://doi.org/10.1016/j.ijpharm.2011.01.030 (2011).

11 Wood, P. A. *et al.* Knowledge-based approaches to co-crystal design. *CrystEngComm* **16**, 5839-5848, doi:10.1039/C4CE00316K (2014).

12 Klímová, K. & Leitner, J. DSC study and phase diagrams calculation of binary systems of paracetamol. *Thermochimica Acta* **550**, 59-64, doi:https://doi.org/10.1016/j.tca.2012.09.024 (2012).

13 Karki, S. *et al.* Improving Mechanical Properties of Crystalline Solids by

Cocrystal Formation: New Compressible Forms of Paracetamol. *Advanced Materials* **21**, 3905-3909, doi:https://doi.org/10.1002/adma.200900533 (2009).
14 Berry, D. J. *et al.* Applying Hot-Stage Microscopy to Co-Crystal Screening: A Study of Nicotinamide with Seven Active Pharmaceutical Ingredients. *Crystal Growth & Design* **8**, 1697-1712, doi:10.1021/cg800035w (2008).
15 Szczepanik, R. Two-and multicomponent, solid-liquid systems formed by aromatic hydrocarbons, anthraquinone, and coal-tar fractions. *Chem. Stosowana Ser. A* **7**, 621-660 (1963).
16 Rice, J. W. & Suuberg, E. M. Thermodynamic study of (anthracene+benzo[a]pyrene) solid mixtures. *The Journal of Chemical Thermodynamics* **42**, 1356-1360, doi:https://doi.org/10.1016/j.jct.2010.05.019 (2010).
17 Linstrom, P. & Mallard, W. (National Institute of Standards and Technology (Gaithersburg, MD 20899), 2003).
18 Swami, A. & Jain, R. Scikit-learn: Machine Learning in Python. *Journal of Machine Learning Research* **12**, 2825-2830 (2013).
19 Devogelaer, J.-J., Meekes, H., Tinnemans, P., Vlieg, E. & de Gelder, R. Co-crystal Prediction by Artificial Neural Networks**. *Angewandte Chemie International Edition* **59**, 21711-21718, doi:https://doi.org/10.1002/anie.202009467 (2020).
20 Abadi, M. *et al. TensorFlow: A system for large-scale machine learning.* (2016).
21 Such, F. P. *et al.* Robust Spatial Filtering With Graph Convolutional Neural Networks. *IEEE J. Sel. Top. Signal Process.* **11**, 884-896, doi:10.1109/jstsp.2017.2726981 (2017).
22 Ioffe, S. & Szegedy, C. Batch Normalization: Accelerating Deep Network Training by Reducing Internal Covariate Shift. (2015).
23 Battaglia, P. W. *et al.* Relational inductive biases, deep learning, and graph networks. *arXiv preprint arXiv:1806.01261* (2018).
24 Gilmer, J., Schoenholz, S. S., Riley, P. F., Vinyals, O. & Dahl, G. E. Neural message passing for quantum chemistry. *arXiv preprint arXiv:1704.01212* (2017).
25 Cho, K., Van Merrienboer, B., Bahdanau, D. & Bengio, Y. On the Properties of Neural Machine Translation: Encoder-Decoder Approaches. *Computer ence* (2014).
26 Vinyals, O., Bengio, S. & Kudlur, M. Order Matters: Sequence to sequence for sets. *Computer ence* (2015).
27 Hochreiter, S. & Schmidhuber, J. Long Short-Term Memory. *Neural Computation* **9**, 1735-1780 (1997).
28 Fey, M. & Lenssen, J. E. Fast graph representation learning with PyTorch Geometric. *arXiv preprint arXiv:1903.02428* (2019).
29 Duvenaud, D. *et al.* in *NIPS.*
30 Altae-Tran, H., Ramsundar, B., Pappu, A. S. & Pande, V. Low Data Drug Discovery with One-Shot Learning. *ACS Central Science* **3**, 283-293,

doi:10.1021/acscentsci.6b00367 (2017).

31 Shahriari, B., Swersky, K., Wang, Z., Adams, R. P. & Freitas, N. d. Taking the Human Out of the Loop: A Review of Bayesian Optimization. *Proceedings of the IEEE* **104**, 148-175, doi:10.1109/JPROC.2015.2494218 (2016).

32 Stokes, J. M. *et al.* A Deep Learning Approach to Antibiotic Discovery. *Cell* **181**, 475-483, doi:https://doi.org/10.1016/j.cell.2020.04.001 (2020).

33 Bergstra, J., Yamins, D. & Cox, D. D. Making a science of model search. *arXiv preprint arXiv:1209.5111* (2012).

34 Ross, S., Lamprou, D. & Douroumis, D. Engineering and manufacturing of pharmaceutical co-crystals: a review of solvent-free manufacturing technologies. *Chemical Communications* **52**, 8772-8786 (2016).

35 Etter, M. C. & Reutzel, S. M. Hydrogen bond directed cocrystallization and molecular recognition properties of acyclic imides. *Journal of the American Chemical Society* **113**, 2586-2598, doi:10.1021/ja00007a037 (1991).

36 Musumeci, D., Hunter, C. A., Prohens, R., Scuderi, S. & McCabe, J. F. Virtual cocrystal screening. *Chemical Science* **2**, 883-890 (2011).

37 Gaussian 09, Revision B.01 (Wallingford, CT, 2009).

38 Lu, T. & Chen, F. Multiwfn: A multifunctional wavefunction analyzer. *Journal of Computational Chemistry* **33**, 580-592, doi:https://doi.org/10.1002/jcc.22885 (2012).

39 Keshavarz, M. H., Zali, A. & Shokrolahi, A. A simple approach for predicting impact sensitivity of polynitroheteroarenes. *Journal of Hazardous Materials* **166**, 1115-1119, doi:https://doi.org/10.1016/j.jhazmat.2008.12.022 (2009).

40 Kamlet, M. J. & Jacobs, S. J. Chemistry of Detonations. I. A Simple Method for Calculating Detonation Properties of C–H–N–O Explosives. *The Journal of Chemical Physics* **48**, 23-35, doi:10.1063/1.1667908 (1968).

41 Fischer, D., Klapötke, T. M., Reymann, M. & Stierstorfer, J. Dense Energetic Nitraminofurazanes. *Chemistry – A European Journal* **20**, 6401-6411, doi:https://doi.org/10.1002/chem.201400362 (2014).

42 Curtiss, L. A., Raghavachari, K., Redfern, P. C. & Pople, J. A. Assessment of Gaussian-2 and density functional theories for the computation of enthalpies of formation. *The Journal of Chemical Physics* **106**, 1063-1079, doi:10.1063/1.473182 (1997).

43 Göbel, M., Karaghiosoff, K., Klapötke, T. M., Piercey, D. G. & Stierstorfer, J. Nitrotetrazolate-2N-oxides and the Strategy of N-Oxide Introduction. *Journal of the American Chemical Society* **132**, 17216-17226, doi:10.1021/ja106892a (2010).

44 Xie, Y. *et al.* A property-oriented adaptive design framework for rapid discovery of energetic molecules based on small-scale labeled datasets. *RSC Advances* **11**, 25764-25776, doi:10.1039/D1RA03715C (2021).
