# Supplementary material for: Coupling complementary strategy to flexible graph neural network for quick discovery of coformer in diverse co-crystal materials
Source: Nat Commun. 2021 Oct 12;12:5950. doi: 10.1038/s41467-021-26226-7 (PMC8511140; doi:10.1038/s41467-021-26226-7)
Supplement: Supplementary file 2 — Description of Additional Supplementary Files [file 41467_2021_26226_MOESM2_ESM.pdf]

Description of Additional Supplementary Files

File Name: Supplementary Data 1
Description: Refcodes of positive cocrystal samples collected from Cambridge Structural Database.

File Name: Supplementary Data 2
Description: The Negative samples collected in this study.
